# The N-terminal domains of NLR immune receptors exhibit structural and functional similarities across divergent plant lineages

**DOI:** 10.1093/plcell/koae113

**Published:** 2024-04-10

**Authors:** Khong-Sam Chia, Jiorgos Kourelis, Albin Teulet, Martin Vickers, Toshiyuki Sakai, Joseph F Walker, Sebastian Schornack, Sophien Kamoun, Philip Carella

**Affiliations:** Cell and Developmental Biology, John Innes Centre, Norwich NR4 7UH, UK; The Sainsbury Laboratory, University of East Anglia, Norwich NR4 7UH, UK; Sainsbury Laboratory, University of Cambridge, Cambridge CB2 1LR, UK; Computational and Systems Biology, John Innes Centre, Norwich NR4 7UH, UK; The Sainsbury Laboratory, University of East Anglia, Norwich NR4 7UH, UK; Department of Biological Sciences, University of Illinois at Chicago, Chicago, IL 60607, USA; Sainsbury Laboratory, University of Cambridge, Cambridge CB2 1LR, UK; The Sainsbury Laboratory, University of East Anglia, Norwich NR4 7UH, UK; Cell and Developmental Biology, John Innes Centre, Norwich NR4 7UH, UK

## Abstract

Nucleotide-binding domain and leucine-rich repeat (NLR) proteins are a prominent class of intracellular immune receptors in plants. However, our understanding of plant NLR structure and function is limited to the evolutionarily young flowering plant clade. Here, we describe an extended spectrum of NLR diversity across divergent plant lineages and demonstrate the structural and functional similarities of N-terminal domains that trigger immune responses. We show that the broadly distributed coiled-coil (CC) and toll/interleukin-1 receptor (TIR) domain families of nonflowering plants retain immune-related functions through translineage activation of cell death in the angiosperm *Nicotiana benthamiana*. We further examined a CC subfamily specific to nonflowering lineages and uncovered an essential N-terminal MAEPL motif that is functionally comparable with motifs in resistosome-forming CC-NLRs. Consistent with a conserved role in immunity, the ectopic activation of CC_MAEPL_ in the nonflowering liverwort *Marchantia polymorpha* led to profound growth inhibition, defense gene activation, and signatures of cell death. Moreover, comparative transcriptomic analyses of CC_MAEPL_ activity delineated a common CC-mediated immune program shared across evolutionarily divergent nonflowering and flowering plants. Collectively, our findings highlight the ancestral nature of NLR-mediated immunity during plant evolution that dates its origin to at least ∼500 million years ago.

## Teaser

Plant immunity mediated by a major class of immune receptors is conserved among flowering and nonflowering plants, and these receptors have domains with similar structures and functions.

## Introduction

Immune receptors play a central role in perceiving and responding to host cell invasion by parasitic organisms. In plants, decades of functional genetic research have solidified the role of nucleotide-binding domain and leucine-rich repeat (NLR) proteins as intracellular resistance (R) receptors that provide robust defenses against pathogen infection (Kourelis and van der Hoorn 2018; van Wersch et al. 2020). Although genomic studies have recently revealed the occurrence of NLRs across a diverse range of land plants and their algae predecessors (Gao et al. 2018; Andolfo et al. 2019; Shao et al. 2019), our understanding of NLR function is limited to the angiosperm lineage (flowering plants). To date, each of the ∼450 experimentally validated NLRs is from model or crop species of flowering plants. Given that angiosperms are a relatively young lineage, our current view of NLR diversity and evolution in plants is narrow. In particular, the extent to which plant NLRs are functionally conserved across a deep macroevolutionary timescale is unclear.

NLRs are modular proteins consisting of an N-terminal domain (NTD), a central NB-ARC domain, and a C-terminal region containing leucine-rich repeats (LRR) or other superstructure-forming repetitive elements (Sarris et al. 2016; Lapin et al. 2022). The NB-ARC domain functions as a switch that controls the “on/off” state of the receptor, whereas the variable NTD defines different NLR classes. In plants, NLR NTDs include the coiled-coil (CC), RPW8-type CC (CC_RPW8_), and toll/interleukin-1 receptor (TIR) domains, whereas metazoan NLRs typically encode N-terminal PYRIN or caspase recruitment domains (CARD) (Lechtenberg et al. 2014; Kim et al. 2016; Kourelis et al. 2021). NLR NTDs are viewed as the “executioner” or “signaling” domain encoding the biochemical activities that lead to immunity. Indeed, ectopic expression of NTDs in planta, either alone or translationally fused to fluorescent proteins like yellow fluorescent protein (YFP), is often sufficient to promote domain self-association and activate plant immune responses (Bernoux et al. 2011; Bentham et al. 2018; Chia and Carella 2023). Typical outputs downstream of NLR NTD activity include defensive hormone accumulation/signaling, transcriptional reprogramming, reactive oxygen species (ROS) accumulation, and in many cases a localized form of programmed cell death known as the hypersensitive response (HR) (Lolle et al. 2020; Dalio et al. 2021).

Structural and biochemical studies have revealed the molecular functions of NLR subtypes. Upon pathogen virulence factor-dependent activation, the Arabidopsis (*Arabidopsis thaliana*) CC-NLR receptor hopZ1-Activated Resistance1 (ZAR1) forms higher-order oligomer complexes (“resistosome”) in which the primary helix of each CC domain 4-helix bundle assembles into a funnel-shaped structure predicted to form pores within the plasma membrane (Wang, Wang, et al. 2019; Wang, Hu, et al. 2019). In support of this idea, activated *Arabidopsis* ZAR1 or wheat (*Triticum aestivum*) Stem rust resistance 35 (Sr35) pentamers were shown to accumulate at lipid bilayers and act as nonselective cation channels in vitro (Bi et al. 2021; Förderer et al. 2022). This paradigm was further confirmed for the CC_RPW8_ domains of *Arabidopsis* NRG1 and ADR1, which exhibited oligomerization-dependent ion channel activity requiring the N-terminal region of the CC_RPW8_ domain (Jacob et al. 2021). In contrast, activation of the *Arabidopsis* TNL receptors Recognition of Peronospora Parasitica1 (RPP1) and Recognition of XopQ1 (ROQ1) induces oligomerization that reconstitutes a TIR holoenzyme complex capable of hydrolyzing NAD^+^ to produce small molecules that bind to ENHANCED DISEASE SUSCEPTIBILITY1 (EDS1) regulatory complexes (Ma et al. 2020; Martin et al. 2020; Huang et al. 2022; Jia et al. 2022). This, in turn, recruits CC_RPW8_-NLR receptors to execute plant immune responses (Wu et al. 2019; Huang et al. 2022; Jia et al. 2022).

In plants, NLRs function at varying levels of connectivity, ranging from standalone “singleton” receptors sufficient to induce NLR-mediated immunity, paired NLRs that distribute perception (sensor) and transduction (helper) activities, and in fortified networks where a minimal set of helper NLRs function alongside an extensive repertoire of sensors (Adachi, Derevnina, et al. 2019). Within this framework, several conceptual models have emerged to explain how NLRs are poised to monitor for pathogen virulence factors (effectors) and/or their activity. While direct interactions between pathogen effectors and plant NLRs can occur, not all effectors directly interact with cognate NLR receptors (van Wersch et al. 2020). In many instances, NLRs monitor (or *guard*) the integrity of key host proteins and activate immunity upon effector-mediated disruption (Van Der Biezen and Jones 1998). NLRs also monitor nonfunctional “decoy” guardees that are often related to functionally relevant host targets (van der Hoorn and Kamoun 2008). Strikingly, such decoy domains are frequently incorporated within NLRs themselves, with the resulting “integrated domain” responsible for effector recognition being genetically fused to the canonical NLR structure (Sarris et al. 2016).

Our understanding of NLR form and function is limited to angiosperms (flowering plants), which are an evolutionarily young lineage that diverged from nonflowering ancestors ∼209 million years ago in the Upper Triassic era (Li et al. 2019). The first land plants evolved from freshwater charophyte algal predecessors over 500 million years ago (Cambrian–Ordovician) and diverged into several key lineages that predate the angiosperms (Morris et al. 2018). This includes the nonvascular seed-free bryophytes (liverworts, hornworts, and mosses), vascular seed-free lineages like lycophytes (clubmosses) and monilophytes (ferns and horsetails), and the seed-bearing but nonflowering gymnosperms (conifers, cycads, ginkgos, and gnetophytes). Genomic data demonstrate that NLRs are present across land plants and in some of their algal predecessors (Gao et al. 2018; Andolfo et al. 2019; Shao et al. 2019). However, functional analyses of NLRs from nonflowering plants are lacking. In particular, the extent to which NLR immune receptors or their N-terminal executioner domains are functionally conserved across the full spectrum of plant evolution is unclear.

In this study, we undertook a comparative macroevolutionary approach to understand the extent to which NLRs are functionally conserved across divergent plant lineages. We first defined the NLR immune receptor repertoires of distantly related plant genomes (algae to angiosperms) for comparison against experimentally validated NLRs (Kourelis et al. 2021; Liu et al. 2021). Phylogenetic analysis of the central NB-ARC coupled with sequence and structural characterization of NTDs revealed the diversity and evolutionary history of NLR subtypes across plant lineages. In planta expression screening of widely distributed (CC and TIR) as well as atypical (hydrolases and kinases) NLR NTDs confirmed their roles in immune-related processes (HR cell death) in *Nicotiana benthamiana*. Further examination of a CC_MAEPL_ domain subtype enriched in nonflowering plants uncovered molecular and mechanistic similarities to angiosperms. Moreover, phenotypic dissection of nonflowering CC_MAEPL_ activity in *Nicotiana* and the model liverwort *Marchantia polymorpha* identified shared aspects of the CC response despite over 450 million years of divergence. Collectively, our data reveal deep evolutionary conservation of NLR immune receptor executioner domains in plants and hints toward an ancestral CC-mediated immune program.

## Results

### Major plant lineages harbor an extended spectrum of NLR immune receptors

**Figure 1.**
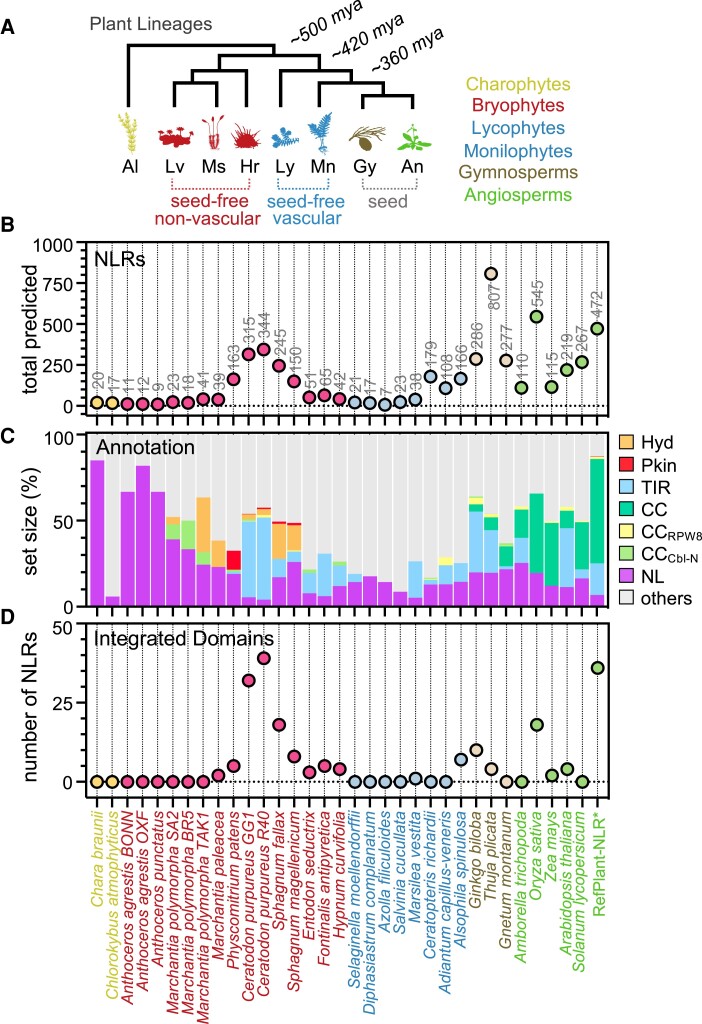
Major plant lineages harbor diverse NLR immune receptors. **A)** Graphical representation of the evolutionary history of major plant lineages that include streptophyte algae (Al), liverworts (Lv), mosses (Ms), hornworts (Hr), lycophytes (Ly), monilophytes (Mn), gymnosperms (Gy), and angiosperms (An). The indicated transitions represent a timescale of millions of years ago (mya) based on previous estimates (Morris et al. 2018). Not to scale. **B)** Total number of NLR-related loci predicted by the NLRtracker annotation tool per species/group. **C)** Diversity of NLR receptor subtypes per species/group as predicted by NLRtracker. Categories are based on predicted NTDs and include TIR-type (TIR), CC-type (CC), RPW8-type (CC_R_), Cbl-N-type (CC_Cbl-N_), hydrolase-type (Hyd), protein kinase-type (Pkn), undefined/minimal NB-ARC-LRR type receptors (NL), and “other” annotation classes (non-NB-ARC-LRRs). **D)** Total number of NLR immune receptor–integrated domains (NLR-IDs) predicted per species/group by NLR tracker.

To identify NLR immune receptors across distantly related green plants, we applied the NLR annotation tool “NLRtracker” (Kourelis et al. 2021) to diverse plant genomes. While our primary focus was on terrestrial seed-free plants (bryophytes, lycophytes, and monilophytes), we included 2 streptophyte algae and 3 gymnosperms (seed-bearing but nonflowering) for comparison alongside angiosperm model plants and experimentally validated NLRs (RefPlantNLR) (Kourelis et al. 2021). Collectively, we queried 33 organisms that capture green lineage diversity across >500 million years of evolutionary divergence, inclusive of freshwater aquatic algae (sister to all terrestrial plants), nonvascular/seed-free bryophytes, vascular but seed-free lycophytes/monilophytes, and nonflowering gymnosperms alongside angiosperm NLRs (Fig. 1A). Our analyses identified NLRs (NB-ARC-containing), degenerate NLRs (partial NB-ARC features), and NLR-associated proteins (RPW8, MLKL, TIR-X, CC-X) in all tested species (Supplementary Data Set 1, see Materials and methods). Amongst our set of distantly related plants, the largest receptor repertoires were observed in mosses (especially *Ceratodon purpureus*; >315), the terrestrial ferns *Ceratopteris richardii* (179) and *Alsophila spinulosa* (166), the gymnosperm *Thuja plicata* (807), and model angiosperms (Fig. 1B).

A survey of NLR subdomain composition revealed a diverse set of immune receptor architectures that included subtypes common to angiosperms (TIR, CC, and CC_RPW8_), with notable expansions of TIR-NLRs in mosses (Fig. 1C). In addition, we identified receptor subtypes enriched within nonflowering lineages, including receptors containing an N-terminal αβ-hydrolase (Hyd-NLRs), protein kinase (Pkn-NLRs), or a Cbl-N domain (4-helix bundle) with homology to the N-terminus of the human proto-oncogene casitas B-lineage lymphoma (CBL). NB-ARC-LRR receptors without annotated N-termini (NL) and NB-ARC proteins with alternative C-terminal repeats (others) were also identified. Lastly, we inventoried NLRs harboring integrated domains (NLR-IDs) at the N or C-terminus of the receptor. This revealed an expansion of NLR-IDs in mosses (*Ceratodon* and *Sphagnum*), the fern *A. spinulosa*, gymnosperms (*Ginkgo* and *Thuja*), and in the flowering plant rice (*Oryza sativa*) (Fig. 1D, Supplementary Data Set 1). Altogether, our analyses confirm the widespread prevalence and diversification of NLRs across distantly related plant lineages.

### NLRs share deep evolutionary ancestry despite NTD diversity

**Figure 2.**
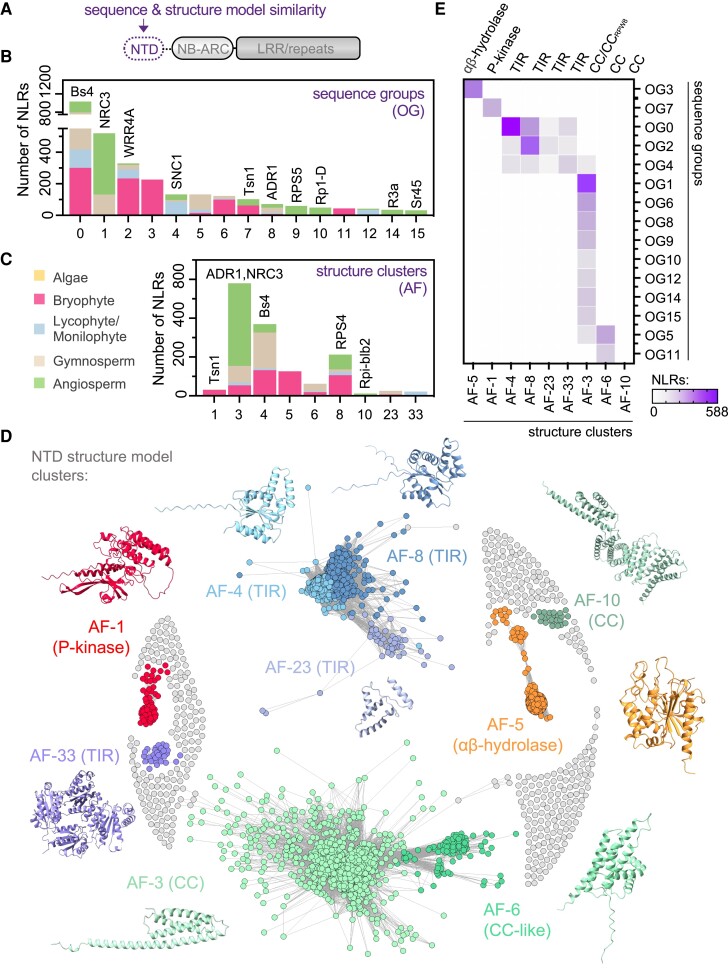
NLR NTDs are structurally conserved across plants. **A)** Schematic overview of the canonical NLR immune receptor structure highlighting the NTD that was characterized by amino acid sequence homology and protein structure similarity. **B)** Distribution of major NLR NTD sequence groups (OG; orthogroups) across NLRs from distantly related plant lineages. Only the most prevalent sequence groups are displayed (at least 30 unique loci across 3 species), with reference NLRs indicated where appropriate. **C)** Distribution of major NLR NTD structure model clusters (AF; AlphaFold2) across NLRs from distantly related plant lineages. Only the most prevalent structure model clusters are displayed (minimum 20 members per community), with reference NLRs indicated where appropriate. **D)** Structure model networking analysis (TM > 0.5) annotated by major NTD clusters. Representative NTDs are displayed next to each major cluster (AF1, TSN1 kinase; AF3, ADR1 CC_RPW8_; AF4, Bs4 TIR; AF5, CepurR40.11G074500.1 αβ-hydrolase; AF6, AagrOXF_evm.model.utg000049l.476.1 CC-like; AF8, RPS4 TIR; AF10, Rpi-blb2 CC; AF23, CepurGG1.12G062000.1 TIR; AF33, Ceric.15G040200.1 TIR). **E)** Overlap between major NLR NTD sequence groups (OG, orthogroups) and NTD structure model similarity clusters (AF, AlphaFold2). Heatmap depicts the number of NLRs shared between sequence and structure groups, with NTD identity indicated.

To better explore plant NLR diversity, we performed a comprehensive interrogation of NTD identities through sequence and structure (AlphaFold2 predicted) similarity approaches (Fig. 2A). First, we assessed the performance of 3 programs to group NLR NTD protein sequences from across plant evolution. Compared against MMSeqs-2 and DIAMOND DeepClust, OrthoFinder generated the greatest number of high-membership sequence groups and was the most inclusive tool for our set of NLR NTDs (Supplementary Fig. S1). In total, 396 sequence groups were produced, of which 15 major groups carried at least 30 unique loci across 3 species (Fig. 2B, Supplementary Data Set 1). Since sequence divergent proteins can adopt similar structures, we also performed structural similarity networking of AlphaFold2-predicted NLR NTD structure models, which produced a total of 58 communities (minimum 2 members) and identified 9 major clusters carrying between 24 and 1,160 members (Fig. 2, C and D, Supplementary Data Set 1). Comparisons between major sequence groups (OGs) and predicted structure clusters (AFs) reliably classified each of the major NLR NTD subtypes in plants (Fig. 2E), including αβ-hydrolases (AF5; OG3), protein kinases (AF1; OG7), TIRs (AF4/8/23/33; OG:0/2/4), and CC-type domains (AF3/6/10; OG1/4/5/6/8/9/10/11/12/14/15). In particular, we observed that 11 major CC domain sequence groups collapsed into 2 principal structure model clusters, demonstrating that these sequence-diverse domains are predicted to adopt comparable protein structures (4 helix bundles).

**Figure 3.**
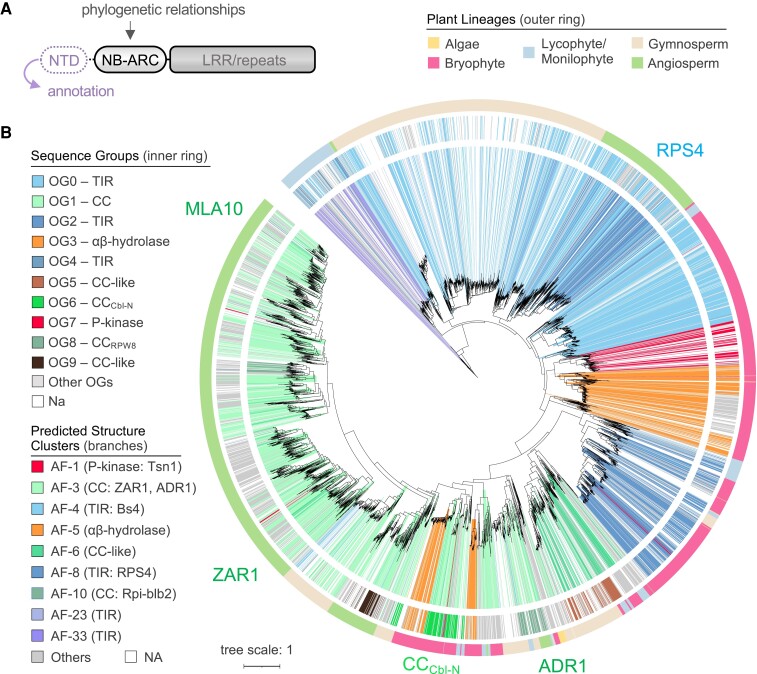
NLRs with diverse NTDs share deep evolutionary ancestry. **A)** Schematic overview of the canonical NLR immune receptor structure highlighting the NTD regions that were used to annotate NLR subclass identity and the central NB-ARC regulatory domain that was subjected to phylogenetic analyses. **B)** Maximum likelihood phylogeny of diverse plant NLRs based on the central NB-ARC regulatory domain. The outer ring represents host lineage/group, the inner ring displays sequence group classification of major NTD OGs, and branch color denotes major structure model clusters (AFs). A representative angiosperm TIR-NLR (RPS4), CC-NLR (ZAR1, MLA10), CC_RPW8_-NLR (ADR1), and the nonflowering CC_Cbl-N_ are indicated. Tree scale = substitutions/site.

To resolve the evolutionary relationships shared between diverse plant NLRs, we performed maximum likelihood phylogenetic analysis of the conserved central NB-ARC region (Fig. 3A). Here, we further benefited from improved NLR subclass annotation provided by NTD sequence grouping and structure model clustering, which allowed us to track NLR architectures over the NB-ARC phylogeny. In general, phylogenetic analysis of the conserved NB-ARC domain combined with NTD annotation demonstrated that the majority of TIR and CC-type NLRs formed separate clades, especially those belonging to angiosperms (Fig. 3B). The annotated phylogeny also highlights the power of structure model clustering to improve NLR classification over a macroevolutionary timescale, as major AF2 clusters covered the vast majority of identified NLRs. We observed a major lineage of CC/RPW8 receptors with representatives across the full spectrum of plant evolution (algae to angiosperms), which included a highly prevalent CC-type sequence group (CC_Cbl-N_) specifically enriched in nonflowering lineages and embedded between NLRs harboring the CC_RPW8_ and angiosperm CC domains (Fig. 3B; Supplementary Fig. S2A). Minor subclades carrying comparatively smaller numbers of moss (*Sphagnum*) hydrolase-NLRs, gymnosperm (*Thuja*) TIR-NLRs, and monocot kinase-NLRs were also embedded within this larger CC/CC_RPW8_ clade, which hints toward the independent acquisition of these architectures from an ancestral CC/CC_RPW8_-type receptor (Supplementary Fig. S2A). By comparison, the majority of plant TIR-NLRs remained separate from CC/CC_RPW8_-NLRs (Fig. 3B; Supplementary Fig. S2B), aside from a prominent subclade of bryophyte TIR-NLRs (AF8 cluster) and an additional subclade of bryophyte CC-like (AF6 cluster) NLRs. The majority of bryophyte hydrolase and kinase-type NLRs were situated closely with bryophyte TIR-NLRs, similar to prior analysis of *M. polymorpha* and *Physcomitrium patens* receptors (Gao et al. 2018; Andolfo et al. 2019). In addition, we identified a subclade of monilophyte NLRs carrying the TIR-like AF33 domain. Receptors harboring the AF23 TIR-like domain were scattered amongst larger AF4 TIR-NLR subclades and may not be a unique subtype of TIR-NLRs. Collectively, these analyses reveal the deep evolutionary history of NB-ARC-containing NLR immune receptors and further highlight the diversity of NTDs encoded by nonflowering plants.

### The NTDs of diverse NLR immune receptors are functional in angiosperms

**Figure 4.**
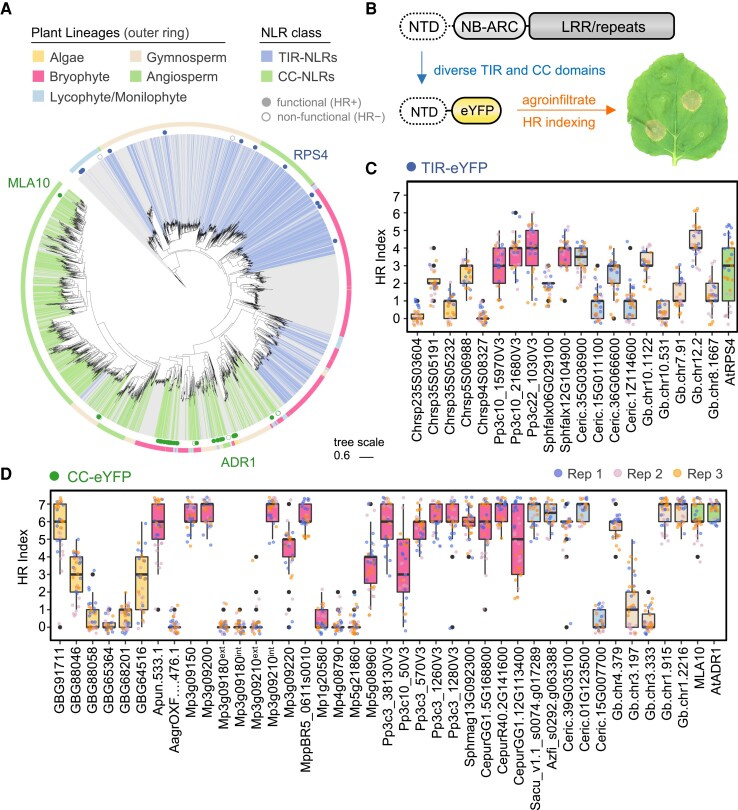
Widely distributed NLR NTDs are functionally transferable across major plant lineages. **A)** Phylogeny of plant NLRs (NB-ARC region) with simplified annotation for CC-NLRs (green, major CC clusters AF3, AF6, and AF10 merged) and TIR-NLRs (blue, major TIR cluster AF4, AF8, AF23, and AF33 merged). The NTDs tested in subsequent experiments are indicated, with functional domains (HR+) indicated by full circles and nonfunctional domains (HR−) indicated by empty circles. **B)** Schematic overview of the experimental design, whereby diverse TIR and CC domains are fused to eYFP, transiently expressed in *N. benthamiana*, and scored for their ability to induce immune-related HR cell death via the HR index (from 0 to 7). Examples of macroscopic cell death phenotypes in an *N. benthamiana* leaf are depicted. **C)** HR cell death caused by the transient expression of TIR-eYFP fusions in *N. benthamiana* leaves. Scoring (HR index) was performed 5 d post agroinfiltration. Box plots represent the median (horizontal line), upper and lower quartiles (boxes), and 1.5× interquartile range (whiskers). Data from 3 independent experimental replicates are shown (*n* ≥ 9 infiltrations per replicate). Information on TIR domain OG/AF identity can be found in Supplementary Data Set 1 (Sheet 7). **D)** HR cell death caused by the transient expression of CC-eYFP (including subfamilies like CC_RPW8_) fusions in *N. benthamiana* leaves. Scoring (HR index) was performed 5 d post agroinfiltration. Box plots represent the median (horizontal line), upper and lower quartiles (boxes), and 1.5× interquartile range (whiskers). Data from 3 independent experimental replicates are shown (*n* ≥ 9 infiltrations per replicate). Information on CC domain OG/AF identity can be found in Supplementary Data Set 1 (Sheet 7).

Our phylogenetic and NTD clustering analyses demonstrate the ubiquitous nature of NLR subtypes across major plant lineages. However, the extent to which divergent NLRs are functionally transferable over a macroevolutionary timescale remains unclear. To address this, we screened the NTDs of diverse TIR and CC-type receptors for their ability to activate HR cell death when transiently expressed as eYFP fusions in the angiosperm *N. benthamiana* (Fig. 4, A and B, Supplementary File 1). We observed moderate-to-strong cell death phenotypes in 11 of 20 TIR domains cloned from nonflowering plants, with those from mosses (*P. patens* and *Sphagnum fallax*), ferns (*C. richardii*), and gymnosperms (*Ginkgo biloba*) exhibiting activity stronger than the *Arabidopsis* RPS4 control (Fig. 4C; Supplementary Data Set 1 and File 1B). TIR fusions were also screened in *Nicotiana tabacum* since TIR-mediated cell death is typically stronger in this species. As anticipated, enhanced TIR-mediated cell death was observed in *N. tabacum* relative to *N. benthamiana* (Supplementary File 1C). Next, we tested whether nonflowering TIRs promote cell death via their enzymatic activity by testing the importance of an essential catalytic glutamic acid (E) residue (Wan et al. 2019). Similar to the angiosperm RPS4 control, HR cell death was abolished in catalytic E-to-A mutants of TIRs derived from the fern *C. richardii* and the gymnosperm *G. biloba* (Supplementary Fig. S3 and File 1D). In comparison with TIRs, a diverse collection of CC-type domains induced strong HR cell death in *N. benthamiana* (Fig. 4D; Supplementary Data Set 1 and File 1E). This covered ∼67% of tested domains from all nonflowering lineages alongside the angiosperm MLA10 (CC) and ADR1 (CC_RPW8_) controls. Predicted CC-type domains from the streptophyte alga *Chara braunii* were used as an outgroup to land plants, with moderate-to-strong activity observed for 3 of 5 tested domains. In addition, we independently assessed each of the 2 CC domains present within predicted *M. polymorpha* tandem CC-CC-NLR receptors (Mp3g09180 and Mp3g09210), identifying HR induction for the internal CC of Mp3g09210 only. Importantly, immunoblots confirmed stable expression of CC/TIR-eYFP fusion proteins, apart from a minority of domains inducing strong cell death phenotypes (Supplementary File 2AB). Together these data suggest that TIR and CC-type domains are functionally conserved across the plant kingdom, highlighting a deep evolutionary origin of NLR immune receptor NTDs in plants.

To begin to explore the atypical NLR NTDs of nonflowering plants, we generated domain-eYFP fusion constructs and tested their ability to activate HR-like responses in *N. benthamiana* (Supplementary Fig. S4A). For the αβ-hydrolases of bryophytes, 2 domains derived from the model liverwort *M. polymorpha* elicited HR-like responses in *N. benthamiana* leaves (Supplementary Fig. S4B and File 1F). To further validate this result, we performed ion leakage assays that are routinely used to monitor membrane integrity and serve as a proxy for NLR-mediated cell death (Hatsugai and Katagiri 2018). Compared against GUS-eYFP, both domains promoted ion leakage at levels comparable with the TIR^RPS4^-eYFP control (Supplementary Fig. S4C). Next, we assessed the functional relevance of N-terminal protein kinase domains of Pkn-NLRs, including that of the wheat Tsn1 receptor (Faris et al. 2010). While a modest yet variable response was observed with a kinase from the moss *P. patens*, a reliable HR was observed for the Tsn1 kinase domain, which was dependent on a previously characterized glycine residue (G29) (Faris et al. 2010) and the catalytic HRD motif (Supplementary Fig. S4B and File 1G). Intriguingly, mild activation of ion leakage was observed for all tested kinases with the exception of the Tsn1^G29E^ mutant (Supplementary Fig. S4C). Immunoblotting demonstrated stable accumulation for the majority of tested constructs (Supplementary File 2C), though reduced but detectable levels of Tsn1^G29E^ were observed. Collectively, these data demonstrate that atypical NLR NTDs are functionally transferable to *N. benthamiana* and are likely involved in mediating immune-like cell death responses across divergent plants.

### Nonflowering plants encode a unique CC domain that harbors a sequence-conserved N-terminal “MAEPL” motif

**Figure 5.**
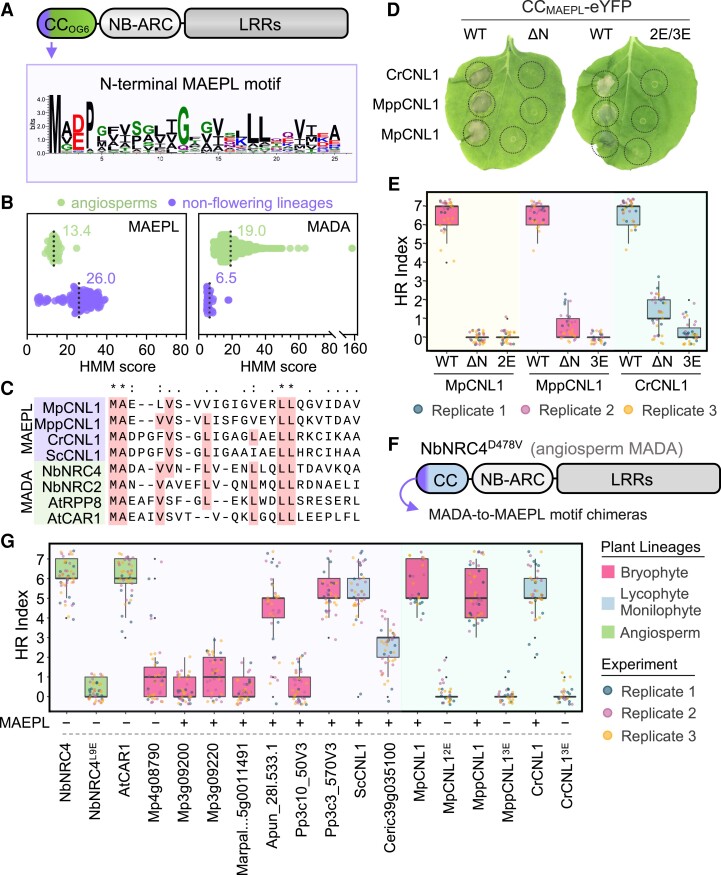
The N-terminal MAEPL motif is essential for nonflowering CC domain activity and is functionally analogous to the angiosperm MADA motif. **A)** Schematic representation of a CC_OG6_-NLR immune receptor. The location of the MAEPL motif on the CC domain is indicated by an arrow, and the consensus amino acid sequence of the motif is illustrated using WebLogo (https://weblogo.berkeley.edu/logo.cgi). **B)** HMM profiling of the N-terminal MAEPL and MADA motifs in nonflowering NLR immune receptors identified in this study (nonflowering) relative to the angiosperm NLR atlas (Liu et al. 2021) (angiosperms). Mean motif scores are indicated on each graph by a numerical value and a dotted line. **C)** Amino acid sequence alignment of MAEPL and MADA motifs in representative CC domains. Conserved residues are indicated by an asterisk (*) above the alignment, similar residues by dots. For nonflowering NLRs, gene symbols correspond to MpCNL1 (*M. polymorpha TAK1*; Mp3g09150), MppCNL1 (*M. polymorpha* ssp. *polymorpha*; MppBR5_0611s0010.1), CrCNL1 (*C. richardii*; Ceric.01G123500.1.p), and ScCNL1 (*Salvinia cucullata*; Sacu_v1.1_s0074.g017289). **D)** Macroscopic HR cell death phenotypes of CC_MAEPL_-eYFP fusions comparing WT domains, N-terminal CC_MAEPL_ truncations (ΔN), and L-to-E CC_MAEPL_ variants (MpCNL1^L16/17E^/2E; MppCNL1^L8/16/17E^/3E; CrCNL1^L10/18/19E^/3E) transiently expressed in *N. benthamiana*. Images were obtained 5 d post agroinfiltration and are representative of 3 independent experiments. **E)** Quantification of HR cell death caused by CC_MAEPL_-eYFP (WT), N-terminal truncations (ΔN), and L-to-E variants (2E/3E) for MpCNL1, MppCNL1, and CrCNL1 domains. Cell death was scored (HR index) 5 d post agroinfiltration. Box plots represent the median (horizontal line), upper and lower quartiles (boxes), and 1.5× interquartile range (whiskers). Data from 3 independent experimental replicates are shown (*n* ≥ 9 infiltrations per replicate). **F)** Graphical representation of the MADA-to-MAEPL N-terminal motif swapping experimental design. An autoactive CC_MADA_-NLR (*N. benthamiana*; NbNRC4^D478V^) was used as a scaffold to assess N-terminal motif competency in *N. benthamiana*. **G)** HR cell death induced by NbNRC4^D478V^-6HA chimeras expressed in *N. benthamiana*. All chimeras were generated using the N-terminal motifs of the indicated CC-NLR receptors (*x* axis). The presence of a MAEPL motif is indicated (+/−). Cell death was scored (HR index) 5 d post agroinfiltration. Box plots represent the median (horizontal line), upper and lower quartiles (boxes), and 1.5× interquartile range (whiskers). Data from 3 independent experimental replicates are shown (*n* ≥ 9 infiltrations per replicate).

The broad distribution of functionally conserved CCs suggests that common principles underpin their activity despite over 500 million years of green plant evolution. To identify conserved and functionally relevant features in divergent CCs, we queried the highly prevalent CC_Cbl-N_ (OG6) domains of nonflowering plants for enriched amino acid sequence motifs using MEME (Multiple EM for Motif Elicitation) (Bailey and Elkan 1994). While this revealed conserved motifs across the entire CC domain (Supplementary File 3), we focused on a motif present at the N-terminus given its high prevalence (80/105) and because similarly situated motifs underpin CC function in angiosperms (Adachi, Contreras, et al. 2019; Jacob et al. 2021). Sequence alignment of this region revealed a conserved consensus motif at the N-terminus that we named “MAEPL” (Fig. 5A; Supplementary File 3). We used this alignment to build a Hidden Markov Model (HMM) profile to further examine MAEPL prevalence across green plant proteomes via the HMMER tool (Eddy 1998). Overall, HMM profiling identified high-scoring MAEPL matches in NLRs predicted by NLRtracker in most nonflowering plants (18/26) and not in angiosperms (Supplementary Fig. S5A). To further extend this comparison, we interrogated the angiosperm NLR atlas (>90,000 NLRs) (Liu et al. 2021) and our combined set of nonflowering lineage NLRs for the presence of MAEPL, which revealed an increased occurrence of high-scoring motifs in nonflowering plants relative to in angiosperms (Fig. 5B; Supplementary Fig. S5B). In contrast, the N-terminal MADA (Adachi, Contreras, et al. 2019) motif of angiosperm CC domains was enriched in angiosperms but not in nonflowering plants (Fig. 5B; Supplementary Fig. S5C). Despite the clear separation of these 2 motifs across flowering and nonflowering plants, alignments comparing MAEPL and MADA reveal intriguing similarities in motif composition (Fig. 5C; Supplementary Fig. S6), including commonly situated leucine residues critical for MADA function in angiosperms (Adachi, Contreras, et al. 2019). Taken together, these results hint that MADA and MAEPL were likely derived from an ancestral CC domain N-terminal motif that diversified independently in flowering and nonflowering plants, respectively.

### MAEPL is required for CC activity and is a functional analog of the angiosperm MADA motif

To determine the functional relevance of MAEPL, we generated N-terminal truncations of 3 CC_MAEPL_-eYFP fusions (MpCNL1, MppCNL1, and CrCNL1) and assessed their ability to activate HR cell death in *N. benthamiana*. In each instance, N-terminal truncations (ΔN) failed to induce cell death, whereas wildtype CC_MAEPL_-eYFP fusions (WT) were fully competent (Fig. 5, D and E; Supplementary File 1H). Next, we generated MAEPL motif variants in which conserved leucine residues (hydrophobic) were replaced by glutamic acid (hydrophilic), a strategy shown to impact MADA function in angiosperms (Adachi, Contreras, et al. 2019). Again, WT CC_MAEPL_-eYFP controls were fully competent while double (2E) or triple (3E) L-to-E mutations abolished HR cell death (Fig. 5, D and E; Supplementary File 1I). Together, these data demonstrate that the MAEPL motif is essential for CC function in a manner analogous to the angiosperm MADA motif. To address this similarity, we tested whether MAEPL motifs from nonflowering CCs can functionally replace MADA in an autoactive variant of the NbNRC4 angiosperm helper CC-NLR (Fig. 5F). The autoactivated NbNRC4^D478V^-6HA receptor retaining its original MADA motif elicited strong HR cell death, while the MADA disrupted L9E variant was nonfunctional (Fig. 5G; Supplementary File 1J). As an additional control, we generated a MADA-to-MADA chimera with the N-terminal motif of Arabidopsis AtCAR1, which effectively rescued HR cell death. Intriguingly, several MAEPL-NbNRC4^D478V^-6HA chimeras exhibited strong HR cell death comparable with NbNRC4^D478V^-6HA (Fig. 5G; Supplementary File 1J), indicating that MAEPL can indeed replace MADA. Importantly, conserved leucine residues within the MAEPL motif were essential for this activity, as the L-to-E variants of MpCNL1, MppCNL1, and CrCNL1 chimeras all failed to elicit an HR (Fig. 5G; Supplementary File 1J). Immunoblots confirmed stable expression for all domains, variants, and chimeras in *N. benthamiana* (Supplementary File 2, D and E).

Emerging data indicate that resistosome-forming CC_MADA_-NLRs associate with the plasma membrane (Adachi, Contreras, et al. 2019; Contreras et al. 2022), which is often observed as discontinuous fluorescent punctae along the membrane in confocal fluorescence microscopy experiments. Similar to angiosperm CC_MADA_-NLRs, the MpCNL1 (Mp3g09150) CC_MAEPL_-eYFP fusion exhibited clear puncta formation that discontinuously localized with a Remorin (REM1.3)-RFP plasma membrane marker, while a GUS-YFP control was nucleocytoplasmic (Supplementary Fig. S7A). Puncta formation was not altered in MpCNL1 2E or 3E CC_MAEPL_ variants (Supplementary Fig. S7A), consistent with observations of stabilized MADA mutant localization in angiosperms (Adachi, Contreras, et al. 2019; Duggan et al. 2021; Contreras et al. 2022). Collectively, these data demonstrate that the divergent MAEPL motif of nonflowering land plants is functionally comparable with the angiosperm MADA.

### CC_MAEPL_ activates immune-like responses in the liverwort *M. polymorpha*

**Figure 6.**
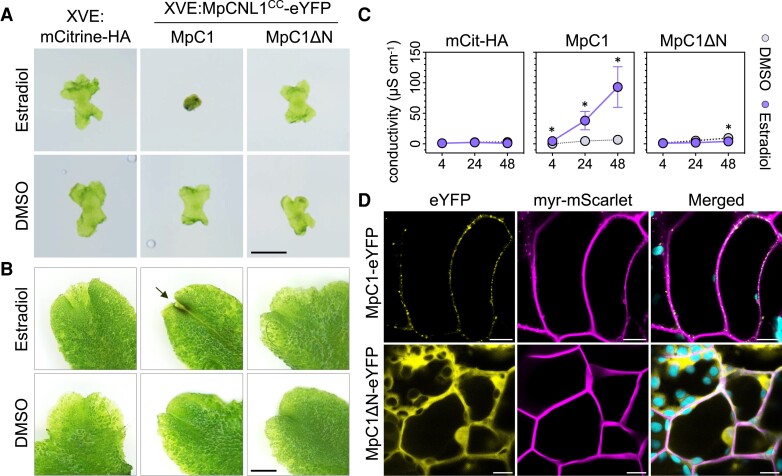
CC_MAEPL_ activates an immune-like response in the liverwort *M. polymorpha*. **A)** Macroscopic phenotypes of *Marchantia* transgenic lines XVE:mCitrine-HA (mCit-HA), XVE:MpCNL1^CC^-eYFP (MpC1, line 1), or the N-terminally truncated XVE:MpCNL1^CCΔN^-eYFP (MpC1ΔN, line 3) grown on estradiol (20 *μ*M) or DMSO (0.1%) control media. Images are representative of growth phenotypes observed in 3 experimental replicates (*n* = 8 plants) at 4 d post plating. Scale bar = 2 mm. **B)** Macroscopic phenotypes of *Marchantia* transgenic lines XVE:mCitrine-HA (mCit-HA), XVE:MpCNL1^CC^-eYFP (MpC1, line 1), or XVE:MpCNL1^CCΔN^-eYFP (MpC1ΔN, line 3) 1 d post vacuum infiltration with estradiol (50 *μ*M) or DMSO (0.25% in water). Images are representative of phenotypes observed in 3 or more experimental replicates (*n* ≥ 8 plants). An arrow indicates tissue darkening at the apical notch of MpC1 liverworts. Scale bar = 2 mm. **C)** Conductivity (*μ*S/cm) of *Marchantia* thalli treated with estradiol (50 *μ*M) or DMSO (0.25%) at 4, 24, and 48 hpi. Statistically significant differences are denoted by an asterisk (*P < 0.05, Student's *t* test). Error bars represent standard deviation of the mean. Data from 3 independent experimental replicates are presented (*n* = 12 plants per experiment). **D)** Confocal fluorescence microscopy shows the localization of MpC1-eYFP and MpC1ΔN-eYFP alongside an myr-mScarlet membrane marker in *M. polymorpha*. Images were acquired 24 h post estradiol treatment (20 *µ*M) in MpC1-eYFP/myr-mScarlet (XVE:MpCNL1^CC^-eYFP/MpEF1a:myr-mScarlet) and MpC1ΔN-eYFP/myr-mScarlet (XVE:MpCNL1^CCΔN^-eYFP/MpEF1a:myr-mScarlet) transgenic lines. Plastid autofluorescence is false-colored in cyan. Scale bars = 10 *µ*m. Images are representative of 3 experimental replicates.

The functional conservation of divergent CC domains in *N. benthamiana* prompted us to examine whether CC_MAEPL_ activates immune responses in nonflowering plants. To address this, we used the model experimental liverwort *M. polymorpha*, a bryophyte species that diverged from *N. benthamiana* over 450 million years ago (Bowman et al. 2022). Using an estradiol-based (XVE) conditional expression system (Furuya et al. 2022), we interrogated the function of the MpCNL1 (Mp3g01950) CC_MAEPL_ domain in the WT TAK1 accession. The importance of the MAEPL motif was assessed by comparing the activity of full-length MpCNL1^CC^-eYFP (MpC1) against an N-terminally truncated MpCNL1^CCΔN^-eYFP variant (MpC1ΔN) and an mCitrine-HA (mCit-HA) control. Severe growth inhibition was specifically observed in MpC1 liverworts cultivated directly on estrogen-supplemented media, which displayed dark pigmentation characteristic of biotic (Carella et al. 2019) and abiotic (Albert et al. 2018) stress in *Marchantia* (Fig. 6A; Supplementary Fig. S8A). In contrast, MpC1ΔN and mCit-HA lines cultivated with estradiol remained healthy, similar to all DMSO controls. These results were further confirmed by exogenous estradiol application in 3-wk-old liverworts, with MpC1 lines exhibiting tissue darkening and brown phenolic-like deposits at apical notches. Again, DMSO controls as well as estrogen-treated MpC1ΔN and mCit-HA remained healthy (Fig. 6B; Supplementary Fig. S8B). Importantly, immunoblotting confirmed estradiol-dependent accumulation of the mCit-HA control and MpCNL1^CC^-eYFP (Supplementary File 2F). In comparison, MpC1ΔN lines exhibited reduced stability in liverwort cells such that full-length MpCNL1^CCΔN^-eYFP fusions were detected alongside eYFP cleavage products (Supplementary File 2F).

Next, we performed trypan blue staining (Ma et al. 2012) to determine whether CC_MAEPL_ causes cell death in liverworts. As outlined in the methods and supporting data, we were unable to obtain conclusive results due to technical difficulties in our liverwort expression system (Supplementary Fig. S8, C and D). We therefore performed ion leakage assays that revealed increasing sample conductivities in estradiol-treated MpC1 tissues from 4 to 48 h post infiltration (hpi), whereas MpC1ΔN and mCit-HA liverworts exposed to estradiol displayed minimal conductivity comparable with DMSO-treated controls (Fig. 6C; Supplementary Fig. S8E). Collectively, these results demonstrate that the CC_MAEPL_ domain activates an immune-like response that overlaps with well-established NLR NTD outputs of flowering plants.

### The CC_MAEPL_ domain forms membrane-localized puncta in liverwort cells

Since CC_MAEPL_-eYFP was observed at membrane-localized puncta in angiosperms, we performed confocal microscopy to examine whether puncta similarly form in nonflowering plants. Confocal microscopy revealed that the MpCNL1^CC^-eYFP fusion formed discontinuous puncta that overlapped with plasma membrane–localized myristoylated-mScarlet (myr-mScarlet) in liverwort cells (Fig. 6D). We also observed MpCNL1^CC^-eYFP signals within intracellular inclusion bodies (Supplementary Fig. S7B). By comparison, N-terminally truncated MpCNL1^CCΔN^-eYFP exhibited nucleocytoplasmic distribution. Together, these data demonstrate that membrane-localized puncta formation is conserved in nonflowering plants and suggest that CC_MAEPL_ targets the membrane for immune-related activity in plant cells.

### CC_MAEPL_ activates liverwort defense gene expression

**Figure 7.**
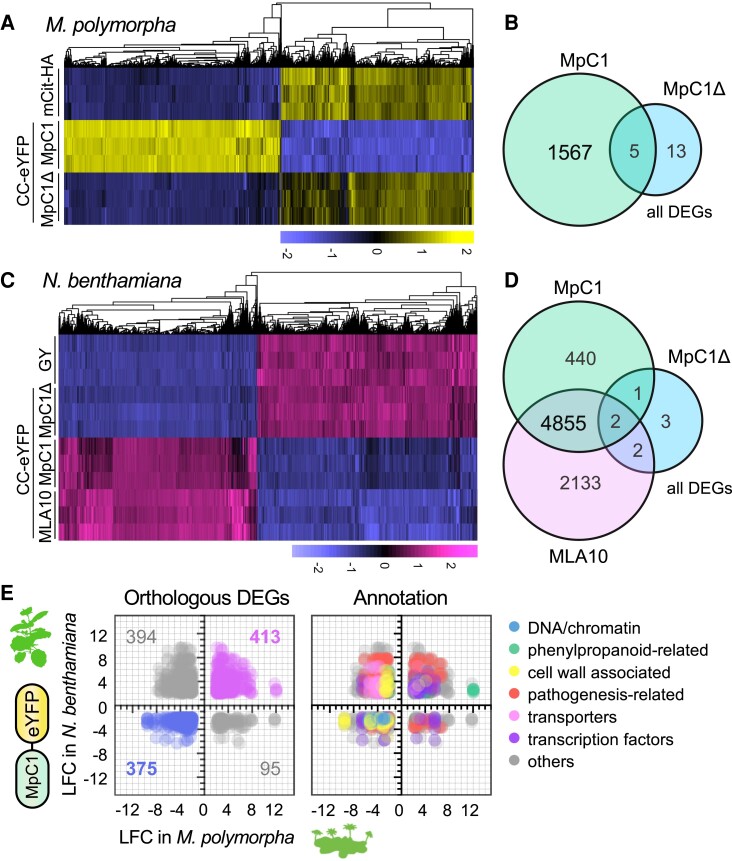
CC_MAEPL_ activates common immune-like transcriptional responses in flowering and nonflowering plants. **A)** Hierarchical clustering of significant differentially expressed genes (DEGs) in mCit-HA (XVE:mCitrine-HA), MpC1 (XVE:MpCNL1^CC^-eYFP, line 1), and MpC1Δ (XVE:MpCNL1^CCΔN^-eYFP, line 3) *M. polymorpha* transgenics 24 h after vacuum infiltration with 20 *μ*M estradiol (adjusted *P*-value < 10^−3^, log fold change (|LFC| ⩾ 2). Variance-stabilized row-centered counts are shown. **B)** Total number of DEGs shared between *M. polymorpha* MpC1 and MpC1Δ transgenic lines. Differential expression is based on comparisons with the mCit-HA control. **C)** Hierarchical clustering of significant DEGs in *N. benthamiana* leaves transiently expressing GY (GUS-YFP), MpC1 (MpCNL1^CC^-eYFP), MpC1Δ (MpCNL1^CCΔN^-eYFP), or the angiosperm CC domain of MLA10 (MLA10^CC^-eYFP) at 24 h post agroinfiltration (adjusted *P*-value < 10^−3^, log fold change (|LFC| ⩾ 2). Variance-stabilized row-centered counts are shown. **D)** Total number of DEGs shared in *N. benthamiana* leaves transiently expressing MLA10, MpC1, or MpC1Δ. Differential expression is based on comparisons with the GUS-YFP control treatment. **E)** Orthology analysis of *Marchantia* and *Nicotiana* MpCNL1^CC^-eYFP transcriptomes. Orthologous genes having an adjusted *P*-value < 10^−3^ and log fold change (LFC) ≥ 2 or ≤−2 were considered. Numbers of DEGs and functional annotation (performed individually for each sector) are indicated.

To understand how the CC_MAEPL_ domain activates immune-related responses in liverworts, we performed RNA-sequencing (RNA-seq) experiments comparing gene expression profiles of estradiol-treated MpC1 (XVE:MpCNL1^CC^-eYFP line 1), MpC1ΔN (XVE:MpCNL1^CCΔN^-eYFP line 3), and mCit-HA (XVE:mCitrine-HA) liverworts at 24 hpi. Transcriptomes of mCit-HA and MpC1ΔN were largely overlapping, whereas MpC1 liverworts displayed a distinct expression profile (Fig. 7A; Supplementary Fig. S9, A and B). Differential expression analysis (log2 fold change [|LFC|] ≥ 2 and adjusted *P* < 10^−3^) comparing MpC1 or MpC1ΔN to the mCit-HA control identified a greater set of differentially expressed genes (DEGs) in MpC1 (1567 DEGs) relative to nonfunctional MpC1ΔN (18 DEGs), with only 5 genes shared between them (Fig. 7B; Supplementary Fig. S9C and Data Set 2). In support of these data, we validated a subset of CC_MAEPL_-responsive genes by RT-qPCR (Supplementary Fig. S9D). Functional enrichment analyses comparing up- and downregulated genes of estradiol-treated MpC1 further defined the CC_MAEPL_ response of liverworts. Terms associated with plant defense responses and biosynthetic activity were enriched in upregulated genes, whereas several terms associated with growth, cellular homeostatic functions, and redox activity were enriched in downregulated genes (Supplementary Data Set 2). Only 18 transcripts were differentially expressed in MpC1ΔN versus mCit-HA controls. Of these, 6 were CC_MAEPL_-NLRs specifically upregulated in MpC1ΔN but not MpC1 liverworts (Supplementary Data Set 2), which hints toward compensatory feedback caused by CC dysfunction. Altogether, RNA-seq analysis supports the idea that CC_MAEPL_ activity prioritizes plant defenses over normal growth and cellular functions in liverworts. We hypothesize this to be a general consequence of CC activity in liverworts since ectopically expressed MLA10^CC^-eYFP (angiosperm CC_MADA-like_) also led to growth inhibition and defense gene activation in *Marchantia* (Supplementary Fig. S10). Such growth–defense tradeoffs are reminiscent of known NLR autoactivity phenotypes in angiosperm models like *Arabidopsis* (van Wersch et al. 2016); however, the extent to which NLR-mediated responses are conserved across distantly related lineages remains unclear.

### CC_MAEPL_ activates common transcriptional responses in flowering and nonflowering plants

The CC_MAEPL_ domain activates immune-like responses in flowering and nonflowering plant species that diverged over 450 million years ago. To understand the extent to which these responses overlap, we compared the MpCNL1^CC^-eYFP activated transcriptome of *Marchantia* to the model angiosperm *N. benthamiana*. To accomplish this, we performed further RNA-seq analyses in *N. benthamiana* leaves transiently expressing MpCNL1^CC^-eYFP (MpC1), MAEPL-truncated MpCNL1^CCΔN^-eYFP (MpC1ΔN), the angiosperm MLA10^CC^-eYFP (MLA10), and a GUS-YFP control. Differential gene expression analyses identified transcriptional shifts upon expression of MpC1 and MLA10 relative to the inactive GUS-YFP and MpC1ΔN controls (Fig. 7C; Supplementary Fig. S9, E and F, and Data S2). The expression profiles of MLA10 and MpC1 generally overlapped, as 2,652 of 3,329 (79.6%) genes activated by MLA10 treatment were similarly activated by MpC1 (Fig. 7D; Supplementary Fig. S9, G and H). This suggests that the divergent CC_MAEPL_ behaves similarly to the angiosperm MLA10 CC domain, consistent with the fact that each domain causes substantial immune-like responses in flowering and nonflowering plants alike.

To begin to unravel the core molecular responses to CC activation in plants, we assessed the extent to which MpCNL1^CC^-eYFP transcriptional responses are shared between *Nicotiana* and *Marchantia*. First, we defined sets of orthologous protein-coding genes (orthogroups) amongst 8 model plants (algae to angiosperms) using OrthoFinder (Emms and Kelly 2019). This revealed a total of 29,268 orthogroups shared amongst distantly related species. We then identified orthologous genes shared between *Marchantia* and *Nicotiana* that were significantly differentially expressed in both plants, revealing 550 orthologous DEGs in *Nicotiana* and 362 orthologous DEGs in *Marchantia* (Supplementary Fig. S11). Amongst this set of shared orthologous DEGs, 62% were commonly up- or downregulated in *Marchantia* and *Nicotiana* (Fig. 7E). In contrast, conflicting expression patterns were observed for orthologous DEGs downregulated in *Marchantia* but upregulated in *Nicotiana*. Through functional enrichment analyses, we identified terms related to DNA-associated machinery represented in genes commonly suppressed upon CC activation, including general transcription factors, replication machinery, and chromatin maintenance genes (Fig. 7E; Supplementary Data Set 2). In contrast, common CC upregulated genes included phenylpropanoid-related enzymes and pathogenesis-related proteins. Conflicting DEGs were enriched in ABC transporters, cell wall proteins, and pathogenesis-related protein families. Despite these intriguing differences, our data hint toward an underlying core program of the plant CC-mediated response that is likely centered on the induction of biochemical immunity alongside the repression of DNA/chromatin homeostasis.

## Discussion

In this study, we demonstrate that NLR immune receptors have retained functionality in their NTDs across 500 million years of plant evolution. We used the angiosperm model *N. benthamiana* to show that CC and TIR domains from algae to angiosperms are capable of translineage immune activation. Given the large evolutionary distances separating these lineages within the plant kingdom, we hypothesize that CC and TIR domains arose early during plant evolution and have retained their biochemical functions throughout the conquest of land.

Our computational analyses demonstrate that sequence-diverse NTDs often adopt similar structural folds (AlphaFold2 predicted). This was particularly evident in highly diverse CC-like domains (4-helix bundles) that generally clustered together in our network-based structural similarity approach. This not only facilitated NLR functional annotation but also provided additional clarity to NLR phylogeny on a macroevolutionary scale. Such structure-guided classification has been an invaluable tool for deep evolutionary comparisons, as evidenced by the identification of sequence-diverse but structurally similar FOLD effectors that are widely distributed across divergent fungal lineages (Teulet et al. 2023; Yu et al. 2023). Looking forward, we propose that structure-guided comparative analyses will be an essential tool for the classification of plant NLRs and may also facilitate cross-kingdom comparisons of NLRs from microbes to metazoans.

We functionally validated the immune activity of a diverse set of CC-type NTDs across plant and algal genomes. The capacity of diverse CCs to activate HR cell death in *N. benthamiana* reveals their ubiquitous role as executors of plant programmed cell death. The translineage activity of CC-like domains from the streptophyte alga *C. braunii* particularly supports this, as it diverged from land plants over 550 to 750 million years ago (Morris et al. 2018). Fungal and metazoan N-terminal 4-helix bundle domains cause cell death in an analogous fashion to plant CC domains (Hildebrand et al. 2014; Daskalov 2016; Bentham et al. 2018; Barragan et al. 2019). Intriguingly, CC domain mechanisms may be widely transferable between kingdoms as cell death was reported in metazoan cells expressing angiosperm CC-NLRs (Jacob et al. 2021; Förderer et al. 2022). While this remains to be explored in further detail, these findings suggest that fundamental biochemical mechanisms may underpin the function of CC-like 4-helix bundles across the tree of life.

The CC_MAEPL_ domain, which generally carries the MAEPL motif at its very N-terminus, is the most common CC subtype in nonflowering lineages. We found that the MAEPL motif is essential for cell death activity in a manner analogous to the MADA motif that occurs in about 20% of angiosperm CC-NLRs (Adachi, Contreras, et al. 2019). HMM profiling of MAEPL and MADA motifs across plant NLRs supports their divergence, with each motif enriched in their respective lineage. Nevertheless, MAEPL motifs from nonflowering plants functionally replaced the MADA motif of the angiosperm helper NLR NbNRC4 despite >450 million years of divergence. We hypothesize that MAEPL and MADA are derived from a common ancestral motif, as phylogenetic analysis of CC_MAEPL_-NLRs and angiosperm CC-NLRs supports common ancestry within the CC/CC_RPw8_ receptor lineage. While each motif has diversified in overall sequence composition, conserved hydrophobic leucine residues are essential for nonflowering MAEPL activity similar to angiosperm MADA motifs (Adachi, Contreras, et al. 2019). The proper placement of hydrophobic residues within N-terminal helices therefore presents as a defining feature of CC-type domains. This implies that sequence variation at the N-terminus can be accommodated provided the appropriate distribution of hydrophobic residues is maintained. In agreement with this hypothesis, CC_RPW8_ domains encode an N-terminal motif that is distinct from angiosperm CCs but retains similarly placed hydrophobic residues (Jacob et al. 2021). It is therefore conceivable that a range of N-terminal motifs have evolved across diverse CC domains of plants, with a CC_RPW8_-like progenitor likely seeding the expansion and divergence of plant CC motifs. Precise biochemical mechanisms underpinning cell death remain to be clarified, though several lines of evidence point toward their involvement in the targeted disruption of cellular membranes and ion channel activity (Adachi, Contreras, et al. 2019; Wang, Wang, et al. 2019; Bi et al. 2021; Maekawa et al. 2022). Further dissection of N-terminal motif evolution is therefore critical for revealing the origin and keystone molecular mechanisms of plant CC-NLR-mediated immunity.

We hypothesize that CC_MAEPL_-NLRs function as singleton or helper NLRs in nonflowering lineages as is suggested for CC_MADA_-NLRs (Adachi, Contreras, et al. 2019). In contrast, a prominent signature of sensor NLRs is the presence of integrated domains that bait pathogen effectors. In angiosperms, NLR-IDs form 5% to 10% of the NLRome (Sarris et al. 2016). Here, we identified a varying range of NLR-IDs across diverse NLR subtypes within ∼4% of the nonflowering plant NLRome. We failed to detect NLR-IDs in CC_MAEPL_-NLRs, further supporting the idea that they function as singleton and/or helper NLRs. The integrated domains of nonflowering plants show similarity to angiosperm NLR-IDs and include protein domains known to be targeted by pathogen effectors like kinases, zinc fingers, thioredoxins, and transcription factors (Sarris et al. 2016; Grund et al. 2019). Moreover, nonflowering NLR-IDs were incorporated into broadly distributed (CC-NLRs and TIR-NLRs) as well as lineage-specific (Hyd-NLRs and Pkn-NLRs) receptors. At present, direct evidence for pathogen-induced NLR-mediated immunity is limited to angiosperms and remains to be discovered in nonflowering lineages. Whether CC_MAEPL_-NLRs serve as helpers to activate immunity upon the perception of effectors by diverse sensor NLRs is an intriguing starting point for the future dissection of NLR-mediated immunity in nonflowering plants.

In *Marchantia*, CC_MAEPL_ accumulation activated an immune-like response that included ion leakage and tissue browning that is often associated with oxidative and/or biotic stress in bryophytes (Ponce de León and Montesano 2013; Clayton et al. 2018; Nelson et al. 2018). Transcriptome analysis revealed an induction of pathogenesis-related and phenylpropanoid biosynthesis genes that are characteristic of induced defenses in *Marchantia* (Carella et al. 2019; Redkar et al. 2022). Further comparisons between *Marchantia* thalli and *Nicotiana* leaves provided insights into common CC responses in divergent plants. Here, the downregulation of cell homeostasis and chromatin-associated machinery was a common feature of CC activation. Intriguingly, this included MiniChromosome Maintenance (MCM) complex genes associated with DNA replication. In metazoans, depletion of MCM abundance is linked to DNA replication failure leading to apoptosis (Ryu et al. 2005; Yang et al. 2020). In plants, MCM depletion causes ovule abortion and activation of the DNA damage response (Holding and Springer 2002; Herridge et al. 2014). Consistent with these observations, CC-NLR (RPM1) activation induces DNA damage in the model angiosperm *A. thaliana* (Rodriguez et al. 2018). Whether this represents a conserved process facilitating immunity or is simply a common consequence of cell death requires further experimentation.

The broadly distributed TIR domain has a deep evolutionary history and contributes to immunity in plants, animals, fungi, oomycetes, bacteria, and archaea (Lapin et al. 2022). Indeed, functional interrogation of TIR domains has demonstrated conserved activity across angiosperms, as monocot TIR-only proteins induce HR cell death in the dicots *N. benthamiana* and *N. tabacum* (Wan et al. 2019; Johanndrees et al. 2022). Moreover, animal and bacterial TIRs display NADase activity, produce immune-related small molecules, and can activate HR cell death in plants (*Nicotiana*) (Wan et al. 2019; Bayless et al. 2022). Conversely, plant TIR domains exhibit conserved NADase activity in *Escherichia coli* similar to prokaryotic TIRs (Wan et al. 2019; Ofir et al. 2021). Our functional interrogation of TIR domains from nonflowering plants and streptophyte algae further confirms wide functional conservation, as their activity in *N. benthamiana* similarly required TIR enzymatic activity. Intriguingly, this occurs even though seed-free plants lack EDS1, the central regulator of TIR activity in flowering plants (Lapin et al. 2020). A recent study demonstrated that EDS1 is not required for the activity of a monocot (maize) TNP-family TIR domain in *Nicotiana* (Johanndrees et al. 2022). Given that TIR-type receptors originate in lineages lacking EDS1, it is likely that EDS1-independent immune responses arose early in the evolution of land plants and algae. Further research exploiting nonflowering model systems enriched with TIRs (mosses) is likely to provide insights into the diversification of TIR-mediated immune mechanisms in plants.

Molecular genetic NLR research has historically focused on a limited group of angiosperm crops and model systems. Here, we took a comparative macroevolutionary approach to broaden our functional understanding of the NLR landscape in diverse plant lineages. We identified structurally and functionally conserved NLR NTDs spanning the full spectrum of plant evolution. Our first look into CC immune function in the divergent model bryophyte *M. polymorpha* revealed similarities with the angiosperm *N. benthamiana* despite over 450 million years of divergence. Together, this suggests that NLRs are a core component of the plant immune system. While TIR and CC-type NLRs are likely to retain the functions of their angiosperm counterparts, nonflowering lineages harbor an untapped diversity of atypical NLRs with NTDs (hydrolases and kinases), C-terminal repeats, and integrated domains. Excitingly, our initial phenotypic survey of atypical NLR N-terminal kinase and hydrolase domains demonstrated their potential to participate in immune-related biochemistry. While the precise structure and biochemical mechanisms underpinning their activity remains to be assessed in detail, research in other systems indeed supports the idea that kinase/hydrolase activity is associated with NLR function (Faris et al. 2010; Heller et al. 2018). The extended set of plant NLRs therefore provides exciting potential to identify mechanisms of plant disease resistance and to further dissect the fundamental aspects of immune receptor form and function. Indeed, conceptually similar studies exploring NLR-like proteins of bacteria are beginning to uncover such mechanisms (Gao et al. 2022). Future studies aimed at exploiting the diverse receptor repertoires of plants and algae are therefore likely to advance our fundamental understanding of NLR-mediated immunity and may ultimately inform efforts to engineer disease resistance mechanisms in crops.

## Materials and methods

### Plant growth details

*M. polymorpha* (TAK1) were cultivated axenically from gemmae and grown under a long-day photoperiod (16 h light; ∼80 *μ*E, NET-55-01-11 LED or Sylvania F36W865 fluorescent) on one-half–strength MS (media (pH 6.7) with B5 vitamins at 20 to 22°C. *N. benthamiana* were grown in soil under controlled conditions with a temperature of 22 °C and a long-day photoperiod (16 h light; 160 to 200 *μ*E, fluorescent—Sylvania F58W/GRO).

### Confocal microscopy

For experiments using *M. polymorpha*, confocal laser scanning microscopy was performed on a Leica TCS SP8 equipped with HyD detectors. A white light laser was used to visualize eYFP (excitation 515 nm) and mScarlet (excitation 570 nm). For experiments using *N. benthamiana*, confocal microscopy was performed on a Zeiss LSM880. Argon ion (457/488/514 nm) and HeNe lasers (594 nm) were used to visualize eYFP (excitation 515 nm) and RFP (excitation 594 nm). We collected images from at least 3 independent plants per experimental replicate. All experiments included at least 3 biological replicates (*N. benthamiana* leaf discs or *M. polymorpha* thalli—with at least 3 different regions of the tissue being observed per replicate), and each experiment was performed at least 3 times with similar results.

### Trypan blue staining

Trypan blue staining was performed on liverwort thalli using the protocol described in Redkar et al. (2022) (Redkar et al. 2022). In our conditions, the differential staining of stressed-vs-control liverworts was particularly difficult to discern since apical notches are easily stained and estradiol induction of CC activity led to tissue darkening and phenolic deposits in this area. Tissue clearing (chloral hydrate solution: 2.5 g per mL in distilled water) of unstained liverworts verified this pattern. Trypan blue staining of heat-killed liverworts and *N. benthamiana* leaves undergoing CC-eYFP-induced HR confirmed the viability of staining solutions. For *N. benthamiana*, trypan blue staining was performed as described in Ma et al. (2012) (Ma et al. 2012).

### Transient Agrobacterium-mediated expression and cell death assays

Transient expression of all constructs in *Nicotiana* was performed by agroinfiltration according to methods described in Adachi et al. (2019) (Adachi, Contreras, et al. 2019). Briefly, expanded leaves of 4-wk-old *Nicotiana* plants were infiltrated with *Agrobacterium tumefaciens* GV3101 carrying binary expression plasmids. *A. tumefaciens* suspensions were prepared in fresh infiltration buffer (10 mM MES-KOH,10 mM MgCl_2_, and 150 mM acetosyringone, pH5.6) and adjusted to OD_600_ = 0.25. This suspension was then mixed in a 1:1 ratio with a suspension of *A. tumefaciens* carrying the p19 silencing suppressor. HR cell death phenotypes were scored on an HR index ranging from 0 (no visible symptoms) to 7 (fully confluent cell death). GUS-eYFP constructs were included in each agroinfiltration experiment as a negative control.

### Estradiol induction and ion leakage assays

Estradiol-inducible gene expression in *Marchantia* was achieved by vacuum infiltration generated within the cavity of a needless 50 mL syringe. A 20 mM stock of estradiol (in 100% DMSO) was used to prepare a 50 or 20 *μ*M working concentration (in water). For mock-treatment controls, we used a comparable 0.25% or 0.1% DMSO solution (*v*/*v* in water). To minimize damage caused by tissue handling, all liverworts used for infiltrations were grown on nylon mesh (Normesh, UK), which allowed easy transfer of liverwort thalli to the syringe for treatment. Once infiltrated, thalli were subsequently transferred to clean petri dishes containing at least 2 layers of prewetted filter paper. Plates were sealed with micropore tape and returned to the appropriate growing condition. Where indicated, estradiol treatment was also performed by culturing liverwort gemmae directly into solid media supplemented with estradiol (20 *μ*M) or DMSO (0.1% *v*/*v*). Ion leakage assays were performed essentially as described in Hatsugai and Katagiri (2018) (Hatsugai and Katagiri 2018). Measurements were performed after 4, 24, and 48 h postharvest with a compact conductivity meter (LAQUAtwin-EC-33, Horiba Scientific). Five leaf discs (4 mm diameter) were harvested from different thalli and immersed in 2 mL ddH_2_O as 1 biological replicate. Each measurement contains 3 biological replicates, and all experiments were performed at least 3 times.

### RNA isolation, cDNA synthesis, and RT-qPCR analysis

Total RNA was extracted from flash-frozen *M. polymorpha* (TAK1) plants that were collected 24 h post mock (0.1% DMSO in water) or estradiol (20 *μ*M) treatment using the Spectrum Plant RNA Extraction Kit (Protocol A) with on-column DNAse treatment following the manufacturer's instructions. cDNA was synthesized using 2 *μ*g of total RNA with SuperScript II reverse transcriptase (Invitrogen) following the manufacturer's instructions. qPCR reactions were performed in a total volume of 10 *μ*L using 2.5 *μ*L of 10×-diluted (in nuclease-free water) cDNA and Roche SYBR mix with the primers listed in Supplementary Data Set 3. The qPCR protocol consisted of an initial denaturation at 95 °C for 5 min followed by 40 cycles of 95 °C for 10 s, 60 °C for 15 s, and 72 °C 15 s on a Roche LightCycler 480 II according to manufacturer's instructions. All qPCR primers were designed using Primer3 (Koressaar and Remm 2007; Untergasser et al. 2012) or were previously published as listed in Supplementary Data Set 3. Specificity was validated by analyzing melt curves after each run. Three independent sample replicates as well as 3 technical replicates per sample were performed at any given time point/treatment. Calculations of expression levels normalized to internal controls (2^−ΔCp^ method), and statistical analyses (ANOVA, Tukey's HSD) were performed using R software, and all graphs were generated in GraphPad Prism (v9.3.1).

### Cloning and *Marchantia* transformation

NbNRC4 chimera constructs were amplified by PCR with primers containing BsaI cloning sites, the appropriate N-terminal motif, and overlapping NbNRC4^D478V^ sequences using a NbNRC4^D478V^-6HA construct (Adachi, Contreras, et al. 2019) as a template (Supplementary Data Set 3). To generate NLR NTD (CC, TIR, Hyd, and Pkn) eYFP fusion constructs, we synthesized codon-optimized domains flanked by BsaI restriction sites (Azenta). CC, TIR, and Pkn were synthesized up to (but not including) NB-ARC start sites, whereas *Marchantia* Hyd domains were synthesized up to the middle of an alpha helix that links hydrolases to the NB-ARC. Synthesized gene fragments, or the GUS control (Addgene #50332), were assembled with pICH85281 (mannopine synthase promoter + W [MasWpro], Addgene #50272), pICSL50005 (YFP, TSL SynBio), pICSL60008 (Arabidopsis heat shock protein terminator [HSPter], TSL SynBio), and the binary vector pICH47742 (Addgene #48001) in a Golden Gate compatible system. To generate *Marchantia* expression vectors, the MpCNL1 (Mp3g01950, 1 to 266 aa) and MpCNL1ΔN (Mp3g01950, 31 to 266 aa) were cloned by PCR (Q5 high-fidelity polymerase, NEB) with attL-containing primers using codon-optimized gene fragments as a template. mCitrine-HA and myr-mScarlet were amplified from template plasmids containing the respective fluorophore: mCitrine/pMpGWB105 (Addgene #68559) and mScarlet:CETN2 (Evangelisti et al. 2019). To generate fluorophore fusions, we used a multistep PCR approach with overlapping primers to generate an HA tag on the C-terminal end of mCitrine or a myristolation sequence at the N-terminus of mScarlet. Amplicons were flanked by attB sites to enable recombination into pDONR221 using BP Clonase II (Invitrogen) following manufacturer instructions. MpCNL1^CC^-eYFP, MpCNL1^CCΔN^-eYFP, MLA10^CC^-eYFP, and mCitrine-HA inducible expression constructs were generated by LR recombination reactions into pMpGWB168 (XVE::GW) (Furuya et al. 2022). The plasma membrane marker construct MpEF1a:myr-mScarlet was generated by LR recombination into pMpGWB303 (Addgene #68631) (Ishizaki et al. 2015). All resulting constructs were transformed into *A. tumefaciens* GV3101 (pMP90) by electroporation. *M. polymorpha* transformation was performed using the *Agrobacterium*-mediated thallus regeneration method (Kubota et al. 2013) in the TAK1 background. Transformants were selected on solid one-half strength MS-B5 media supplemented with cefotaxime (125 *μ*g/mL) and hygromycin B (15 to 25 *μ*g/mL) or chlorsulfuron (0.5 to 1 *μ*M). Stable transgenic plants were obtained by propagating gemmae from T1 thalli. All experiments were performed in the G2 (second asexual/gemmae generation) or subsequent generations.

### Protein immunoblotting

Protein samples were prepared from 4 tissue discs (8 mm diameter) sampled from *N. benthamiana* leaves at 1 or 2 d after agroinfiltration and were homogenized in 100 *μ*L of 2× SDS loading buffer (0.1 M Tris-HCl, 0.2 M DTT, 4.0% [*w*/*v*] SDS, 3 mM bromophenol blue, 2 M glycerol). Protein samples from *Marchantia* were prepared from 5 tissue discs (4 mm diameter) with 50 *μ*L of 2× SDS loading buffer. Immunoblotting was performed with HA-probe (F-7) horseradish peroxidase (HRP) (sc-7392 HRP, Santa Cruz Biotech, 1:5,000 dilution) or primary anti-GFP antibody (11814460001, Roche, 1:2,500 dilution) combined with secondary HRP-linked antimouse IgG (NXA931-1ML, Amersham, 1:5,000 dilution). Total protein loading was visualized by staining nitrocellulose membranes with Ponceau S solution (Sigma-Aldrich, P7170).

### Library preparation and sequencing

mRNA from *M. polymorpha* transgenics 24 h post estradiol treatment or *N. benthamiana* leaves 24 h post agroinfiltration were purified from DNAse-treated total RNA (prepared as described above) using Poly(A) selection and then fragmented (at least 3 independently treated plants collected per sample replicate). cDNA library preparation was performed with the TruSeq RNA Sample Preparation Kit (Illumina, United States) according to manufacturer's instructions. Sequencing of each sample (in triplicate) was performed on the Illumina NovaSeq in 150 paired-end mode. Demultiplexed samples were used for subsequent analyses. All raw fastq data are accessible at http://www.ncbi.nlm.nih.gov/sra/ under the accession number PRJNA881591.

### Expression, orthology, and functional enrichment analyses

We first analyzed raw sequencing reads with FastQC for quality control (https://www.bioinformatics.babraham.ac.uk/projects/fastqc/). Reads were then aligned back to the appropriate genome (*M. polymorpha* v5.1, https://marchantia.info/download/tak1v5.1/; *N. benthamiana* draft genome v3.5, https://www.nbenth.com/) using HiSAT2 (Kim et al. 2019). We used featureCounts (Liao et al. 2014) to obtain raw counts using only uniquely mapped and properly paired reads. DEGs were identified with DESeq2 (Love et al. 2014)⁠ following pairwise comparisons between controls (mCitrine-HA control for *Marchantia* experiments; GUS-YFP control for *N. benthamiana* experiments*)* and the indicated treatment conditions. We focused only on DEGs with a strict cutoff (absolute LFC [log2 fold change] ≥ 2 and adjusted *P*-value ≤ 10^−3^) when performing hierarchical clustering of samples. Heatmaps were generated with R pheatmap using variance-stabilized counts median-centered by gene. Functional enrichment analyses (Fisher's exact test) were performed using the MBEX online tool (https://marchantia.info/mbex/) (Kawamura et al. 2022) with a significance cutoff of false discovery rate (FDR)-corrected *P*-values ≤ 0.05.

We used OrthoFinder (OrthoFinder-2.5.4) (Emms and Kelly 2019) to reconstruct orthologous protein groups (orthogroups) shared between distantly related organisms. For comparative transcriptomics, we performed OrthoFinder on the proteomes of *C. braunii*, *Anthoceros agrestis* (OXF), *C. purpureus* (GG1), *C. richardii*, *G. biloba*, *A. thaliana*, *M. polymorpha* (TAK1), and *N. benthamiana*. All orthogroups shared between *M. polymorpha* and *N. benthamiana* were considered in our analyses, and visualization was performed using GraphPad Prism9 software.

### NLR prediction, phylogenetics, protein sequence, and structure model analyses

We used NLRtracker (Kourelis et al. 2021) to identify and annotate NLRs in the genome annotations listed in Supplementary Data Set 1 (Sheet 1). NLRtracker identifies NLRs (containing an NB-ARC domain), degenerate NLRs (potential receptors carrying a P-loop harboring nucleotide hydrolase), and NLR-associated proteins such as RPW8, TX (TIR domain alongside P-loop harboring nucleotide hydrolase), CC-X (CC domain alongside P-loop carrying nucleotide hydrolase), and MLKL (mixed lineage kinase-like pseudokinase). An updated description of the NLRtracker pipeline can be found online (https://github.com/slt666666/NLRtracker). For phylogenetic analysis, we performed the following cleaning procedure 13 times before the full maximum likelihood inference to help account for misidentified homology and potential errors that may arise while inferring a phylogeny with thousands of tips. The sequences were aligned using Pasta (Mirarab et al. 2015) v1.9.0 with the datatype “--protein” option and default parameters. The alignments were then cleaned for a minimum of 10% occupancy using the *pxclsq* program from the Phyx v1.2 package (Brown et al. 2017). The relationships were then inferred using a fast maximum likelihood as implemented in the program IQ-TREE (Nguyen et al. 2015) v1.6.12, with the “--fast” option and the “JTT + F + G4” that was inferred to be the best model using ModelFinder (Kalyaanamoorthy et al. 2017). The resulting homolog tree was then cleaned from errors in homology following the Yang and Smith procedure (Yang and Smith 2014). All branches that are 1.0 subs/site or longer (absolute cutoff) and all branches that are 0.5 subs/site and at least 10× longer than their sister branch (relative) were removed from further analyses. After 13 rounds of cleaning, 252 nonredundant NB-ARCs were removed from our analysis (Supplementary Data Set 1, Sheet 10) and the sequence data was aligned and cleaned using the same procedure as above. The relationships of 4624 remaining sequences were inferred using the full maximum likelihood search as implemented in IQ-TREE v1.6.12 with the “JTT + F + G4” model of evolution and 1,000 ultrafast bootstrap (Hoang et al. 2018) replicates. The resulting tree was again trimmed using the same absolute and relative branch length cutoffs (terminal branches only). All subsequent tree rendering was performed using TVBOT (https://www.chiplot.online/tvbot.html), and a high-resolution phylogeny containing NLR identifiers is provided in Supplementary File 4.

Sequence-based analysis of NLR NTDs (regions upstream of predicted NB-ARCs) was performed using MMseqs2 (Steinegger and Söding 2017), DIAMOND DeepClust (Buchfink et al. 2023), and OrthoFinder (Emms and Kelly 2019). For MMseqs2 and DIAMOND DeepClust, protein sequences were clustered using 50% identity and 50% coverage thresholds through the MPI bioinformatics toolkit (https://toolkit.tuebingen.mpg.de/). OrthoFinder was implemented using default parameters. Structure model predictions and network-based similarity clustering were performed essentially as previously described (Teulet et al. 2023) with slight modifications. First, we implemented ParaFold (Zhong et al. 2022) to facilitate large-scale structure model predictions via AlphaFold2 (Jumper et al. 2021). In total, 5 predicted structure models were generated and the best ranked (ranked_0, determined by pLDDT scores) was selected for downstream analyses (models available at Zenodo, doi: https://doi.org/10.5281/zenodo.8356791). To determine structural similarities between NLR NTD structure models, we generated an all-by-all pairwise comparisons through Foldseek (Van Kempen et al. 2023) using a normalized TM-score. Significant structural similarity was considered for domains with a normalized TM-score ≥ 0.5. Next, the structural comparison was subjected to network analyses using the R package igraph (https://igraph.org/r/) and structure model similarity clustering was conducted by the Louvian method of community detection (Blondel et al. 2008) through igraph.

To identify conserved amino acid motifs in the nonflowering CC_OG6_ clade, we subjected an amino acid sequence alignment of CC_OG6_ domain sequences to MEME analysis (Bailey and Elkan 1994) with the parameters “0 or 1 occurrence per sequence, top 10 motifs.” MEME detected the N-terminal MAEPL motif in several CC_OG6_ domains; therefore, we curated a more specific alignment containing these N-terminal sequences to build a HMM profile of the nonflowering “MAEPL” motif using the “hmmbuild” function in HMMER (version 3.3.2) (Eddy 1998). Upon calibration with “hmmcalibrate,” we tested the Nterm_MAEPL.hmm profile in the proteomes of major plant lineage representatives listed in Supplementary Data Set 1. We further compared the Nterm_MAEPL.hmm with the angiosperm MADA.hmm profile (Adachi, Contreras, et al. 2019) across all plant proteomes and NLRomes used in our study in addition to the angiosperm NLR atlas (https://biobigdata.nju.edu.cn/ANNA/) (Liu et al. 2021). All motif scans were performed using the following template line of code “hmmsearch --max-o output.txt Motif.hmm Proteome.fasta.”

Supporting files and raw data related to NLR prediction, NB-ARC phylogenetic analysis, NTD sequence/structural analyses, N-terminal motif HMM profiling, and orthology analyses (RNA-seq-related) are deposited at Zenodo doi: https://doi.org/10.5281/zenodo.8356791.

### Statistics

Statistical details of experiments can be found in the corresponding figure legends. Here, the identity of the statistical tests used, the exact value of *n* (i.e. number of independently infected liverworts), and dispersion and precision measures are given (error bars represent mean ± Sd, *P*-value cutoffs, etc.). All statistical analyses for transcriptomic and proteomic analyses are described in the methods detailed above. Statistical analysis of RT-qPCR expression data is described in figure legends and was performed using R. Student's *t* tests (2-tailed) were performed on ion leakage data using Microsoft Excel or GraphPad Prism. All values and *P*-values of statistical tests can be found in Supplementary Data Set 4.

### Accession numbers

ADR1 (AT1G33560), CAR1 (AT1G50180), Bs4 (NP_001316454.1), CrCNL1, Ceric.01G123500.1.p), EDS1 (AT3G48090), MpCNL1 (Mp3g09150), MppCNL1 (MppBR5_0611s0010.1), NbNRC4 (QER78241.1), NRG1 (AT5G66900), REM1.3 (AT2G45820), ROQ1 (ATD14363.1), Rpi-blb2 (AAZ95005.1), RPP1 (AT3G44480), RPS4 (AT5G45250), ScCNL1 (Sacu_v1.1_s0074.g017289), Sr35 (AGP75918.1), Tsn1 (ADH59451.1), ZAR1 (AT3G50950).

## Supplementary Material

koae113_Supplementary_Data

## Data Availability

All relevant accession numbers for raw data are provided in the manuscript.

## Supplementary data

The following materials are available in the online version of this article.

**Supplementary Figure S1.** Sequence analysis of NLR NTDs.

**Supplementary Figure S2.** Prominent clades of plant NLRs.

**Supplementary Figure S3.** TIRs of nonflowering plants require catalytic glutamic acid to promote cell death.

**Supplementary Figure S4.** Atypical NLR NTDs activate immune-like responses in *N. benthamiana*.

**Supplementary Figure S5.** MAEPL motif occurrence across major plant lineages.

**Supplementary Figure S6.** MAEPL and MADA N-terminal motif sequence comparison and phylogeny.

**Supplementary Figure S7.** Subcellular localization of CC_MAEPL_ in *Nicotiana* and *Marchantia*.

**Supplementary Figure S8.** Characterization of the CC_MAEPL_ response in *Marchantia*.

**Supplementary Figure S9.** Transcriptomic analysis of CC_MAEPL_ activity in *Marchantia* and *Nicotiana*.

**Supplementary Figure S10.** The angiosperm MADA-like MLA10^CC^ domain provokes immune-like responses in *Marchantia*.

**Supplementary Figure S11.** Orthologous and DEGs used for comparative transcriptomics.

**Supplementary Data Set 1.** Identification and characterization of diverse plant NLRs.

**Supplementary Data Set 2.** RNA-seq analysis of CC_MAEPL_ activity in *Marchantia* and *Nicotiana*.

**Supplementary Data Set 3.** Oligonucleotide primers and synthetic gene fragments used in this study.

**Supplementary Data Set 4.** Values and statistics.

**Supplementary File 1.** Cell death phenotypes of NLR NTDs in *Nicotiana*.

**Supplementary File 2.** Protein immunoblotting.

**Supplementary File 3.** MAEPL motif discovery.

**Supplementary File 4.** Annotated phylogeny of plant NLRs.

## Dive Curated Terms

The following phenotypic, genotypic, and functional terms are of significance to the work described in this paper:

RPM1 Gramene: AT3G07040
RPM1 Araport: AT3G07040

MpCNL1 Gramene: Mp3g09150
MpCNL1 Araport: Mp3g09150
