# Supplementary material for: The N-terminal domains of NLR immune receptors exhibit structural and functional similarities across divergent plant lineages
Source: Plant Cell. 2024 Apr 10;36(7):2491–511. doi: 10.1093/plcell/koae113 (PMC11218826; doi:10.1093/plcell/koae113)
Supplement: koae113_Supplementary_Data [file koae113_supplementary_data.zip › Supplemental Data.pdf]

A.

	MMseqs2	D-DClust	Orthofinder
Total Sequence Groups	430	306	396
Largest group (no. loci)	195	304	1032
Groups with <10 loci	367	237	342
Groups with ≥10 loci	63	69	54
Mean size (groups ≥10 loci)	29	36	66

B.

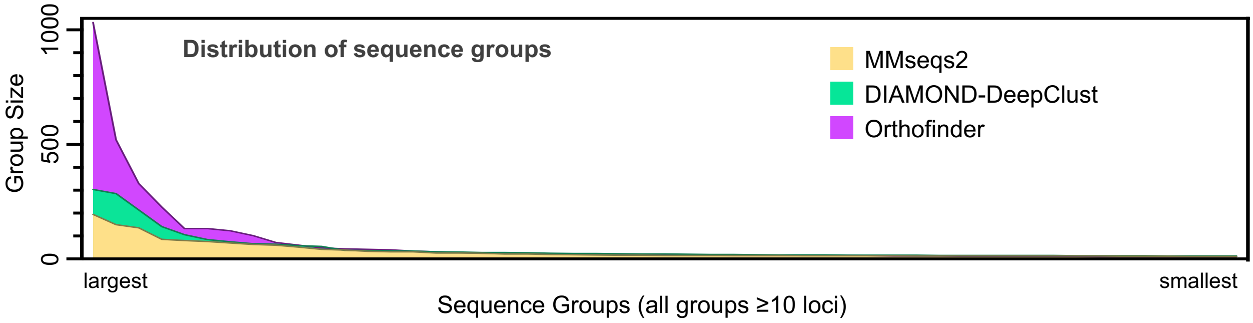

Supplemental Figure S1. Sequence analysis of NLR N-terminal domains. Supports Figure 2
(A) Overall statistics of the protein sequence grouping programs MMseqs2, DIAMOND-DeepClust (D-Dclust), and orthofinder.
(B) Distribution of all sequence groups containing at least 10 loci ordered from largest to smallest along the X-axis.

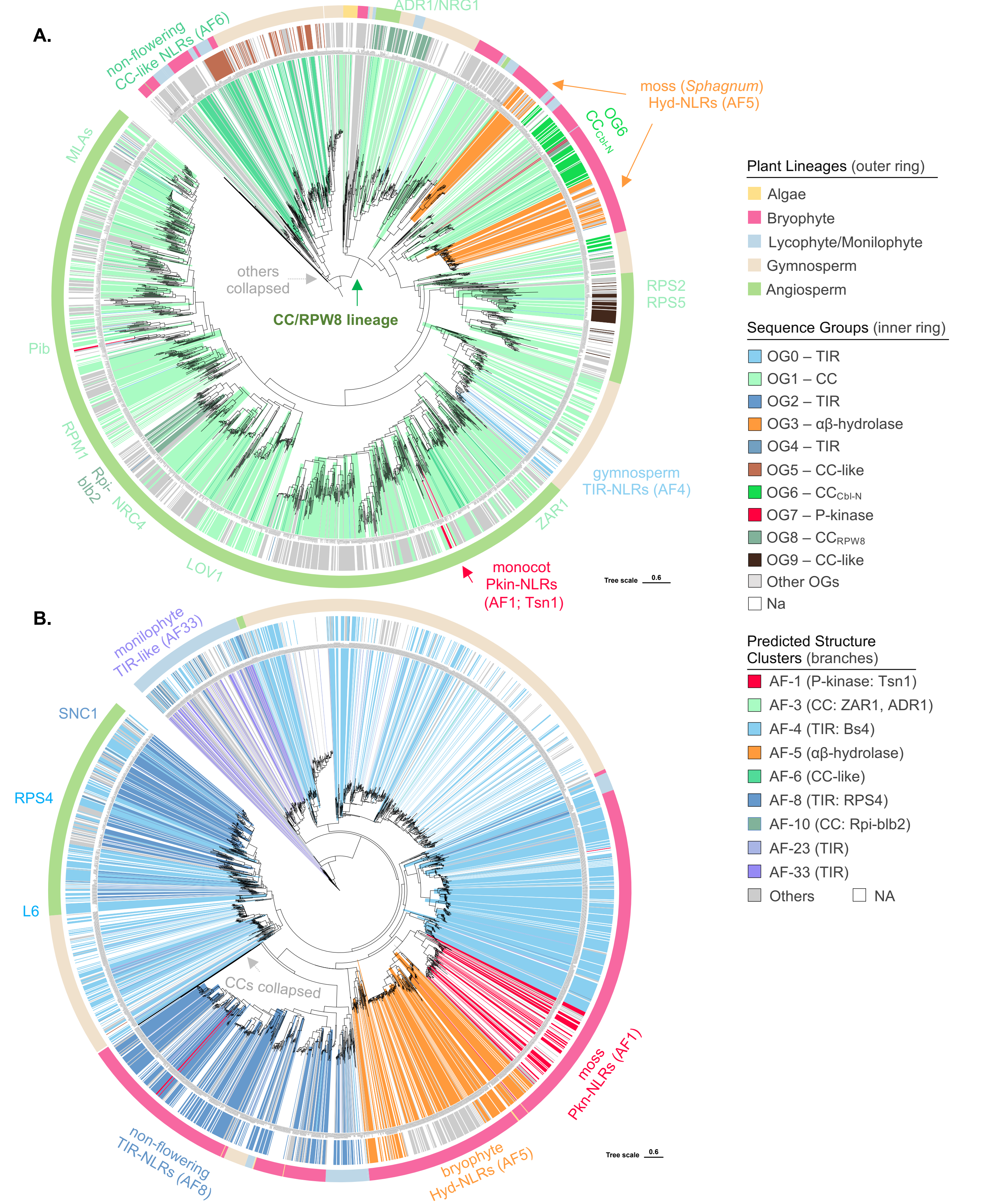

Supplemental Figure S2. Prominent clades of plant NLRs. Supports Figure 3

(A) Highlighting of CC/RPW8-NLRs from the maximum likelihood phylogeny of diverse plant NLRs based on the central NB-ARC regulatory domain (derived from Figure 3). Coloration of the outer ring represents host lineage/group, the inner ring displays sequence group classification of major N-terminal domain OGs, and branch color denotes major structure model clusters (AFs). NLRs outside the main CC/RPW8 lineage (green arrow) were collapsed, with exception of the AF6 subclade enriched in CC-like immune receptors from non-flowering plants. The approximate locations of key subclades and functionally validated NLRs are highlighted. Tree scale = substitutions/site.

(B) Highlighting of TIR-NLRs and other subclasses from the maximum likelihood phylogeny of diverse plant NLRs based on the central NB-ARC regulatory domain (derived from Figure 3). The collapsed CC/RPW8 lineage is indicated by a dashed grey arrow. The approximate position of key subclades and functionally validated NLRS are indicated.

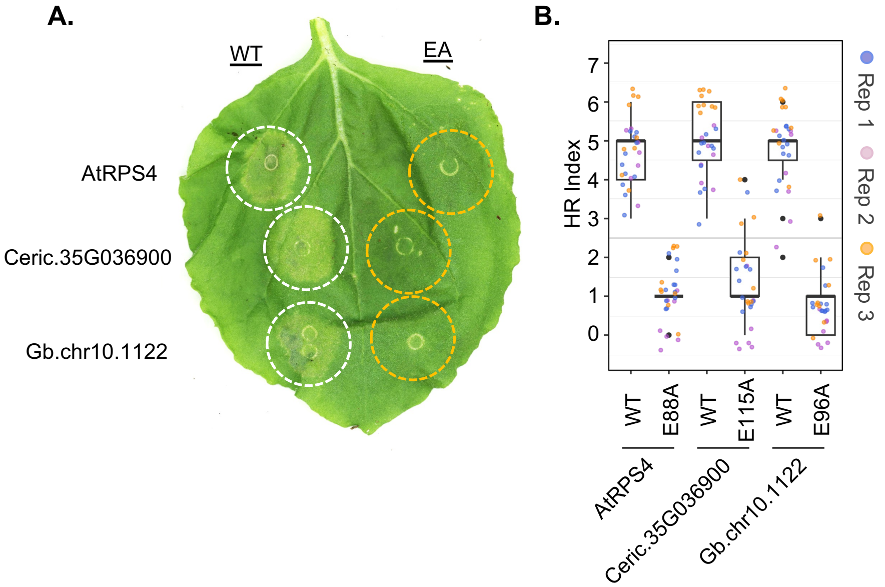

Supplemental Figure S3. TIRs of non-flowering plants require catalytic glutamic acid to promote cell death. Supports Figure 4

(A) Macroscopic HR cell death phenotypes of TIR-eYFP fusions comparing wild-type domains (WT), and catalytic EA mutants (AtRSP4^{E88A}; Ceric.35G036900^{E115A}; Gb.chr10.112^{E96A}) transiently expressed in N. benthamiana. Images were obtained 7 days post agroinfiltration and are representative of 3 independent experiments.

(B) Quantification of HR cell death caused by TIR-eYFP (WT) or EA variants. Cell death was scored (HR index) 5 days post agroinfiltration. Data from three independent experimental replicates are shown (n ≥ 9 infiltrations per replicate).

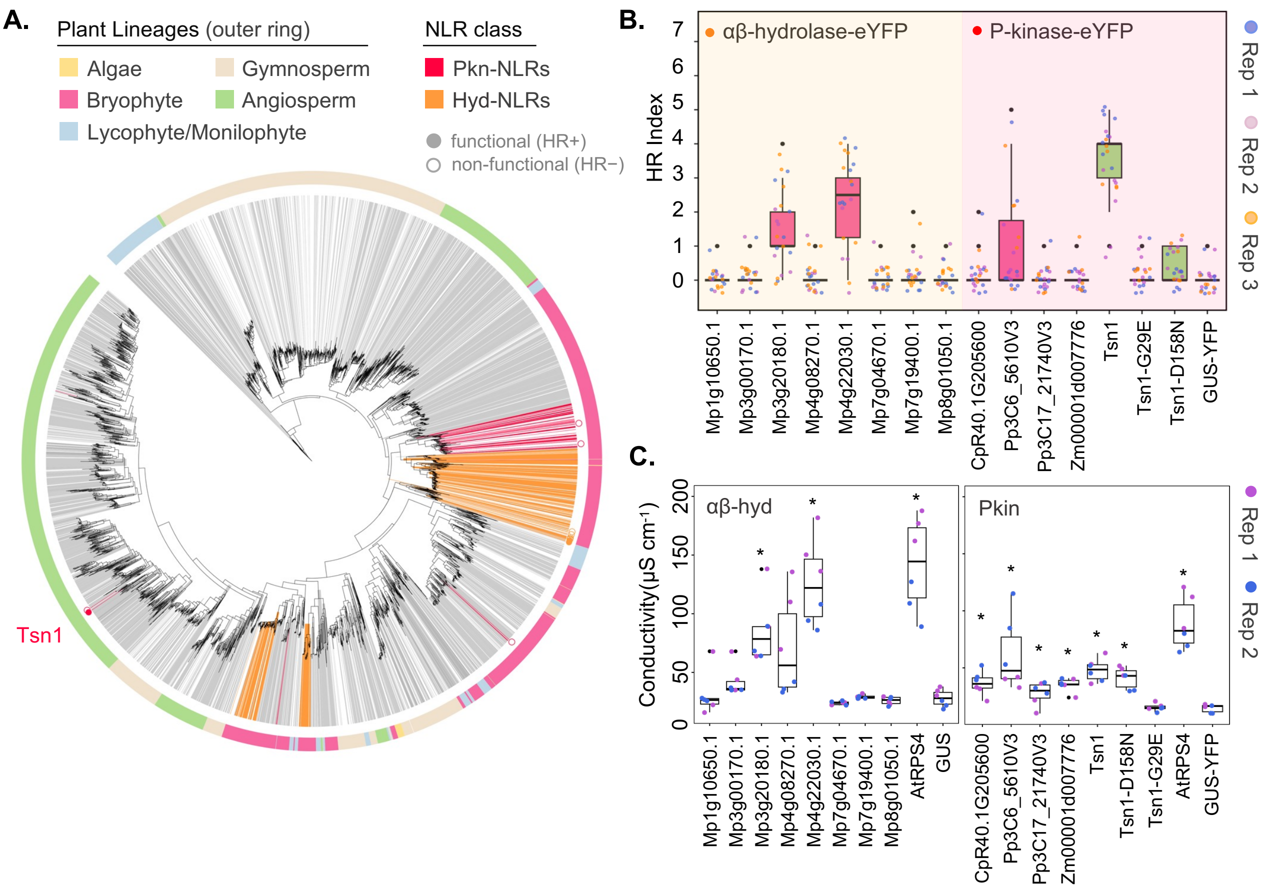

Supplemental Figure S4. Atypical NLR N-terminal domains activate immune-like responses in *N. benthamiana* Supports Figure 4

(A) Phylogeny of plant NLRs (NB-ARC region) with simplified annotation for Pkin-NLRs (red; Protein kinase NLRs) and αβ-Hyd-NLRs (orange; αβ-hydrolase NLRs). The N-terminal domains tested in subsequent experiments are indicated, with functional domains (HR+) indicated by full circles and non-functional domains (HR−) indicated by empty circles.

(B) HR cell death caused by the transient expression of Pkin-eYFP and αβ-Hyd-eYFP fusions in *N. benthamiana* leaves. Scoring (HR index) was performed 5 days post agroinfiltration, Data from for three independent experimental replicates are shown (n ≥ 9 infiltrations per replicate). Box plots represent the median (horizontal line), upper and lower quartiles (boxes) and 1.5x interquartile range (whiskers). Information on atypical NLR N-terminal domain OG/AF identity can be found in Supplementary Data Set S1 (sheet 7). Related to Figure 4.

(C) Conductivity of *Nicotiana benthamiana* leaves transiently expressing the indicated constructs at 5 days post infiltration (hpi). Statistically significant differences are denoted by an asterisk (* $p < 0.05$, Student's t-test – compared against GUS-eYFP control). Error bars represent standard deviation of the mean. Data from two independent experimental replicates is presented.

A.

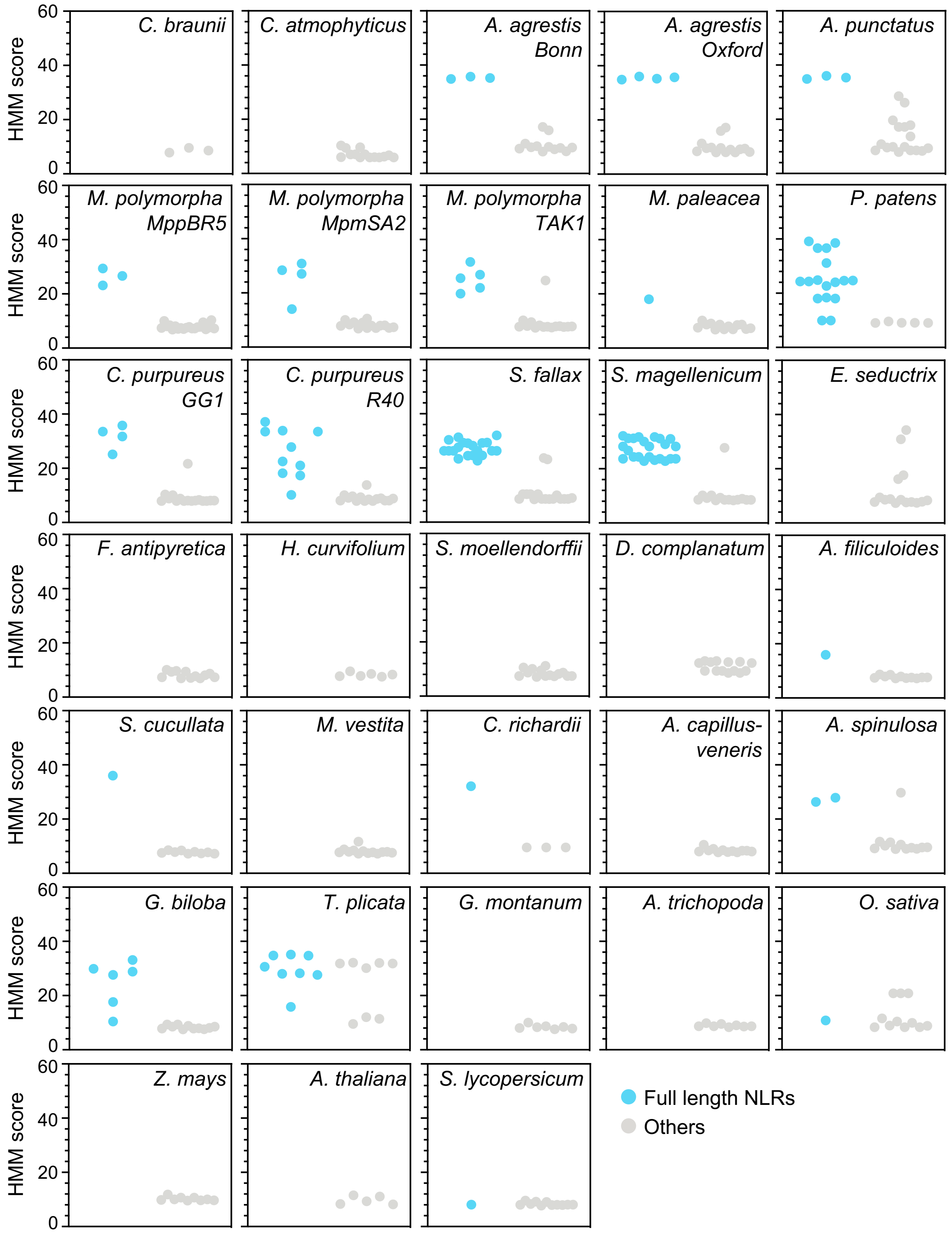

B.

NLR test set	MAEPL	Total NLRs	Prevalence (%)
Angiosperm atlas	49	91,291	0.05
Non-flowering plants	124	4,402	2.82

C.

NLR test set	MADA	Total NLRs	Prevalence (%)
Angiosperm atlas	2979	91,291	3.26
Non-flowering plants	36	4,402	0.82

Supplemental Figure S5. MAEPL motif occurrence across major plant lineages. Supports Figure 5.
(A) MAEPL motif occurrence in full length NLRs (predicted by NLR tracker) relative to other (non-NLR) proteins as identified through HMM profiling (hidden Markov model) across organisms (proteomes) used in this study.
(B) MAEPL motif prevalence in non-flowering plants relative to angiosperms. Angiosperm NLRs were queried using the angiosperm NLR atlas (ANNA).
(C) MADA motif prevalence in non-flowering plants relative to angiosperms. Angiosperm NLRs were queried using the angiosperm NLR atlas (ANNA).

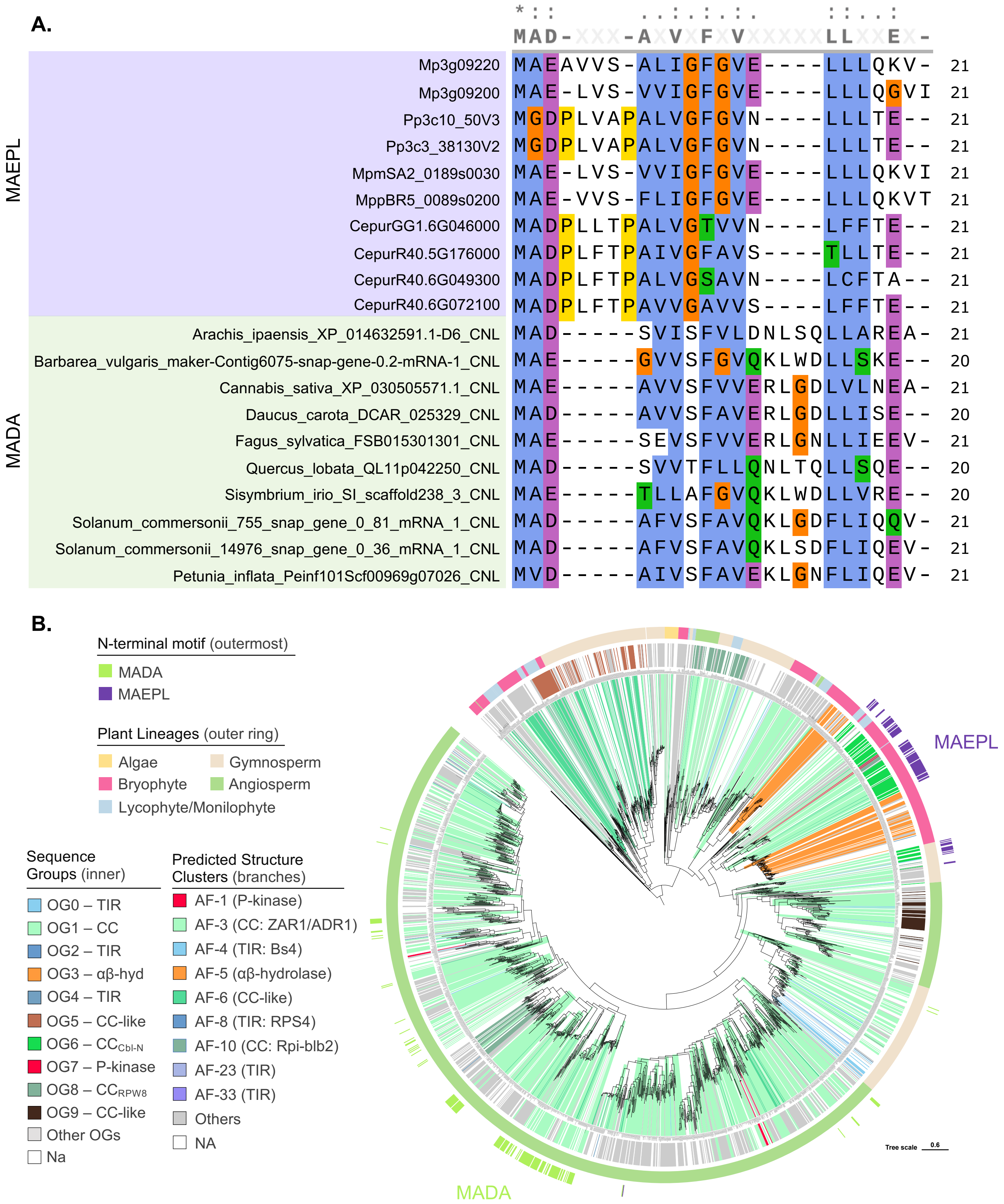

Supplemental Figure S6. MAEPL and MADA N-terminal motif sequence comparison and phylogeny
(A) Amino acid sequence alignment of high-scoring MAEPL motifs (non-flowering OG6-type CC domains) and MADA motifs (queried against CC domains in the angiosperm NLR atlas). Alignments were performed using MAFFT in the SnapGene tool (v6.0.2). The consensus sequence shown represents residues present in >50% of all sequences. Coloring is based on amino acid residue properties + conservation (ClustalX).
(B) NLR phylogeny (based on NB-ARC region of the CC/RPW8-lineage) displaying the occurrence of MAEPL and MADA containing CC-NLRs (derived from Supplementary Fig. S2A). Plant lineages (inner ring), N-terminal domain structure-model clusters (AF2 clusters; branch colors), and MADA/MAEPL identity (outer ring) are labelled on the phylogeny. Tree scale = substitutions/site.

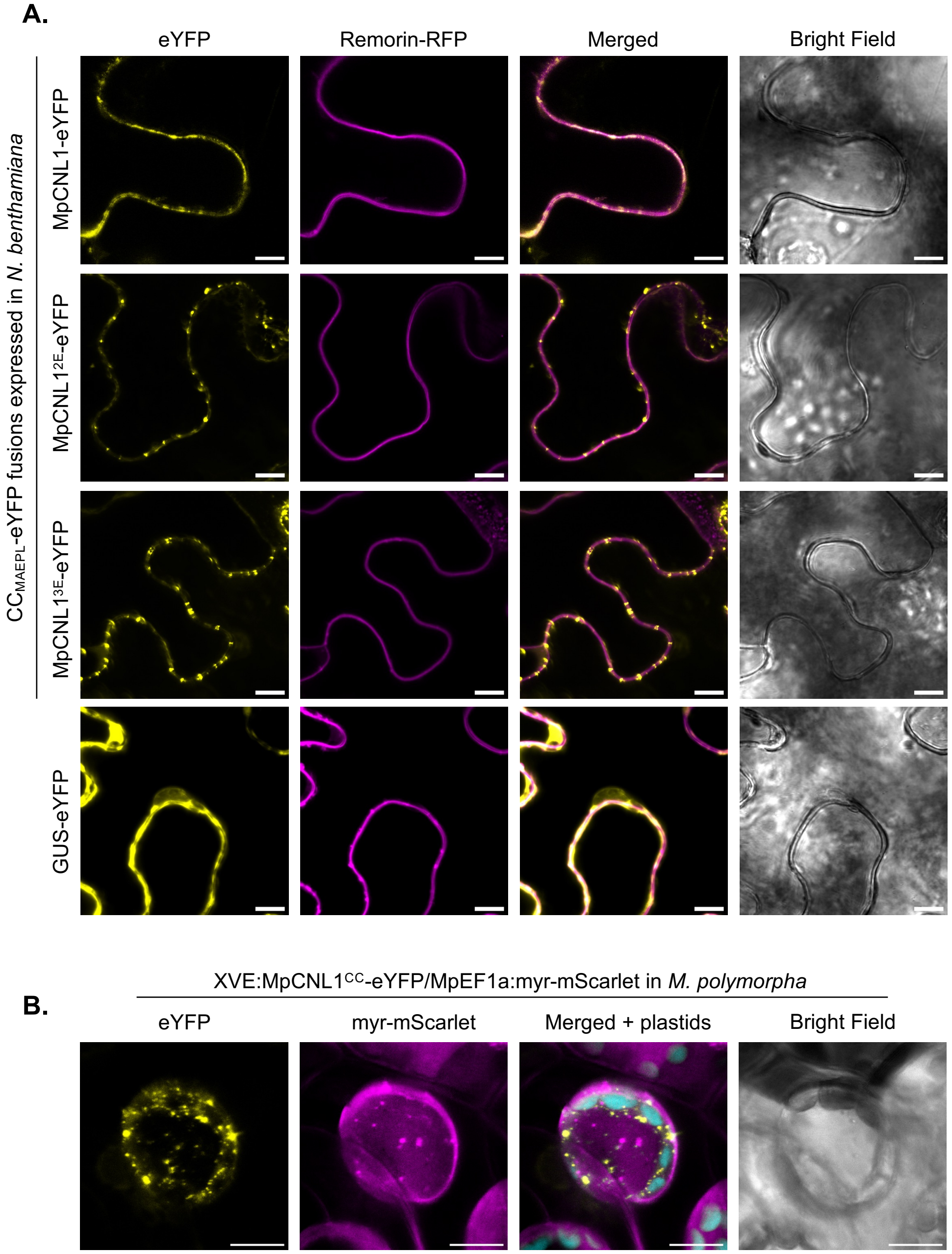

Supplemental Figure S7. Subcellular localization of CC_{MAEPL} in *Nicotiana* and *Marchantia*.
Supports Figures 5 and 6
(A) Confocal fluorescence microscopy demonstrating localization of MpCNL1^{CC}-eYFP fusions and MAEPL motif variants (2E = L16/17E; 3E = L4/16/17E) and a GUS-eYFP control in *N. benthamiana* leaves. The REM1.3-RFP construct was co-infiltrated in combination with each construct to label the plasma membrane. Images were obtained approximately 24 hours post agroinfiltration of *N. benthamiana* leaves. Scale bars = 10 µm. Images are representative of 3 experimental replicates.
(B) Confocal fluorescence microscopy (Z-stack projection) demonstrating localization of MpCNL1^{CC}-eYFP (MpC1) alongside a myristolated-mScarlet (membrane marker) in the *Marchantia polymorpha* XVE:MpCNL1^{CC}-eYFP/MpEF1a:myr-mScarlet transgenic. Images were acquired 24 hours post estradiol treatment (20 µM) in liverwort thalli. Plastid autofluorescence is false-colored in cyan. Scale bars = 10 µm. Images are representative of 3 experimental replicates.

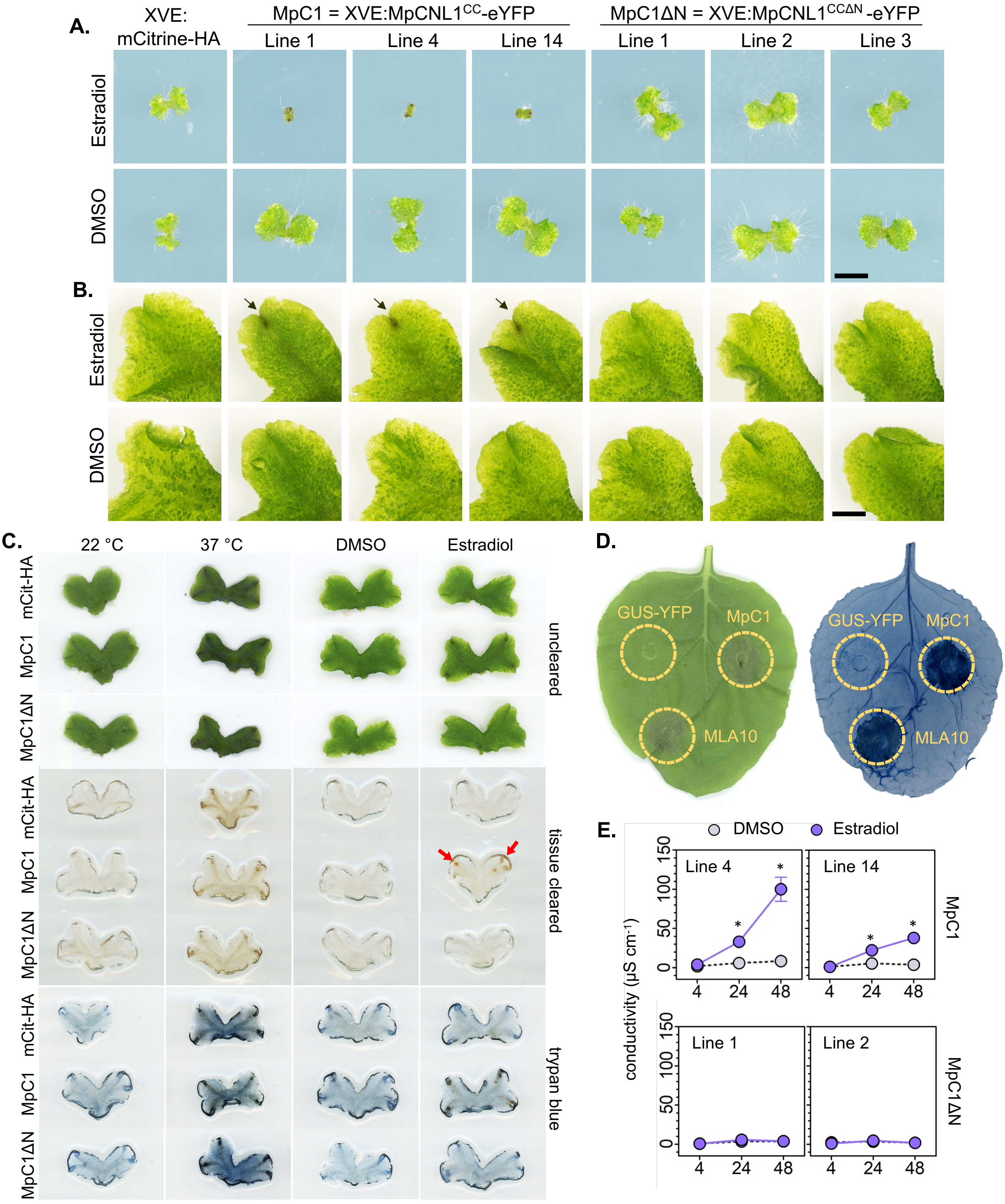

Supplemental Figure S8. Characterization of the CC$_{MAEPL}$ response in *Marchantia*. Supports Figure 6

(A) Macroscopic phenotypes of *Marchantia* transgenic lines XVE:mCitrine-HA (mCit-HA), XVE:MpCNL1^{CC}-eYFP (MpC1 lines 1, 4, and 14), or the N-terminally truncated XVE:MpCNL1^{CCΔN}-eYFP (MpC1ΔN lines 1, 2, and 3) grown with estradiol (20 µM) or DMSO (0.1%) control media. Images are representative of phenotypes observed in 3 experimental replicates (n= 8 plants) at 4 days post plating. Scale bar = 25 mm. (B) Macroscopic phenotypes of *Marchantia* transgenic lines (listed above) at 1 day post infiltration with estradiol (50 µM) or a DMSO control (0.25% in water). Images are representative of phenotypes observed in 3 experimental replicates (n= 8 plants). An arrow indicates tissue darkening at the apical notch of MpC1 liverworts. Scale bar = 25 mm.

(C) Macroscopic phenotypes and trypan blue staining of Marchantia thalli (mCit-HA, MpC1-1, MpC1ΔN-3) subjected to heat-stress (37 °C) or estradiol induction (50 µM). DMSO or 22 °C treatments were included as negative controls. Images display phenotypes 1 day post treatment in uncleared, tissue cleared (chloral hydrate), and trypan blue stained thalli. Images are representative of 3 replicates. Arrows indicate tissue darkening upon estradiol treatment.

(D) Cell death phenotypes (Macroscopic HR and trypan blue staining) 2 days post agroinfiltration of *N. benthamiana* leaves with MpCNL1^{CC}-eYFP (MpC1) and MLA10^{CC}-eYFP (MLA10). GUS-YFP is included as a negative control.

(E) Conductivity (µS cm^{-1}) of *Marchantia* thalli treated with estradiol (50 µM) or DMSO (0.25%) at 4, 24, and 48 hours post infiltration (hpi). Statistically significant differences are denoted by an asterisk (* $p < 0.01$, Student's t-test). Error bars represent standard deviation of the mean. Data from three independent experimental replicates is presented (n=12 plants per experiment).

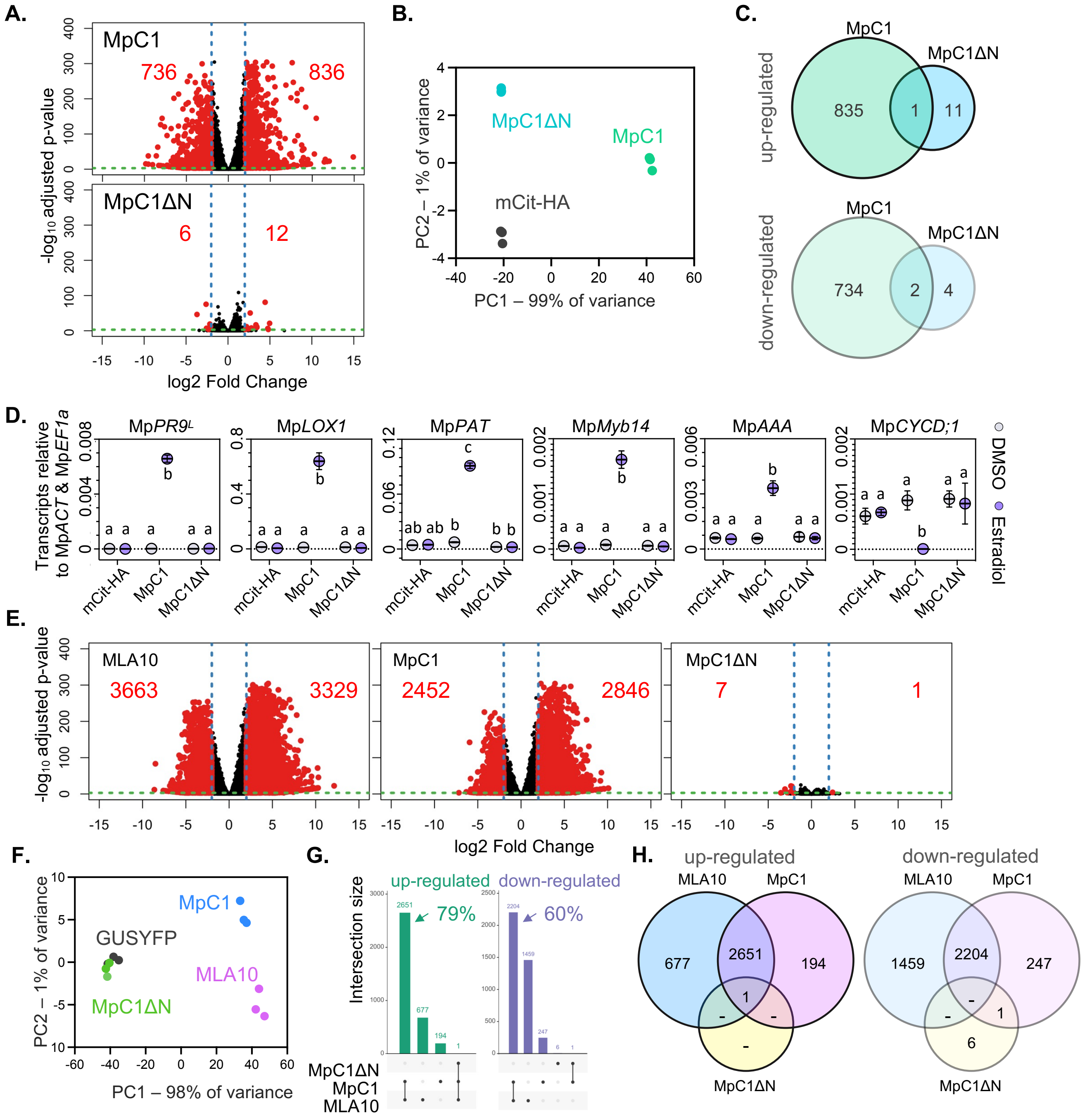

Supplemental Figure S9. Transcriptomic analysis of CC$_{MAEPL}$ activity in *Marchantia* and *Nicotiana*. Supports Figure 7

(A) Volcano plots displaying pairwise differential expression analysis of estradiol-treated (50 µM) MpC1 (XVE:MpCNL1^{CC}-eYFP line1) or MpC1ΔN (XVE:MpCNL1^{CCΔN}-eYFP line 3) versus mCit-HA (XVE:mCitrine-HA) *Marchantia* thalli 24 hours post infiltration. Red dots represent significantly differentially expressed genes. (B) Principal component analysis (PCA) of *Marchantia* transcriptome replicates. (C) Venn diagrams depicting shared/specific differentially expressed genes (DEGs) between treatments in *Marchantia.* (D) RT-qPCR validation of CC marker genes in *Marchantia.* Mp*PR9^L*, Pathogenesis-related 9^L Mp5g21510; Mp*LOX1*, Lipoxygenase Mp2g00660; Mp*PAT*, *Patatin-like phospholipase* Mp5g21760; Mp*Myb14*, R2R3 Myb transcription factor Mp5g19050; Mp*AAA*, Cell death-related AAA-ATPase Mp7g18570; Mp*CYCD;1*, D-type cyclin Mp8g17230) 24 hours after infiltration with DMSO (0.1%) or estradiol (20 µM). Expression values are displayed relative to internal Mp*ACT* and Mp*EF1a* controls. Different letters signify statistically significant differences in transcript abundance (ANOVA, Tukey's HSD, $p < 0.05$). Values represent the mean +/- standard deviation of three biological replicates (n = 6). This experiment was performed three times with similar results. (E) Volcano plots displaying pairwise differential expression analysis of MpC1 (MpCNL1^{CC}-eYFP), MpC1ΔN (MpCNL1^{CCΔN}-eYFP), or MLA10 (MLA10^{CC}-eYFP) versus the GUS-YFP control 24 hours post agro-infiltration in *N. benthamiana* leaves. Red dots represent significantly differentially expressed genes. **(F)** Principal component analysis (PCA) of *Nicotiana* transcriptome replicates. **(G)** UpSet plots showing shared and treatment-specific differentially regulated genes in *N. benthamiana*. Arrows indicate the percentage of DEGs shared between MpC1 and MLA10 treatments. (H) Venn diagrams depicting overlap in differentially expressed genes (DEGs) between treatments in *N. benthamiana.*

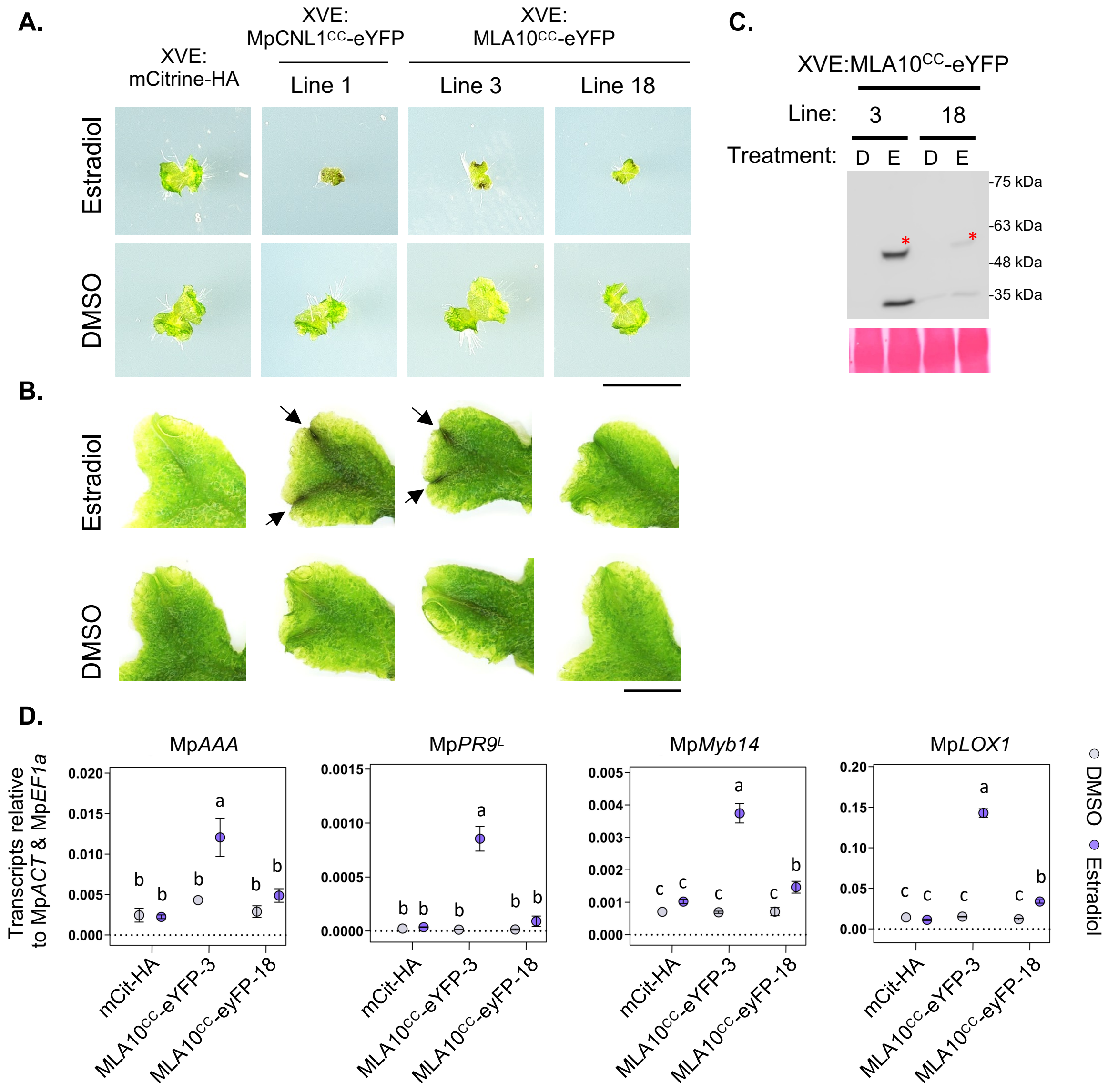

Supplemental Figure S10. The angiosperm MADA-like MLA10^{CC} domain provokes immune-like responses in *Marchantia.* Supports Figures 6 and 7

(A) Macroscopic phenotypes of *M. polymorpha* XVE:MLA10^{CC}-eYFP transgenics (lines 3 and 8) relative to mCitrine-HA (mCit-HA) and XVE:MpCNL1^{CC}-eYFP (MpC1 line 1) controls grown with estradiol (20 µM) or DMSO (0.1%) control media. Images are representative of phenotypes observed in 3 experimental replicates (n= 8 plants) at 7 days post plating. Scale bar = 50 mm.

(B) Macroscopic phenotypes of *Marchantia* transgenic lines (listed above) at 3 day post infiltration with estradiol (50 µM) or a DMSO control (0.25% in water). Images are representative of phenotypes observed in 3 experimental replicates (n= 4 plants). An arrow indicates tissue darkening at apical notches. Scale bar = 50 mm.

(C) Immunoblots of MLA10^{CC}-eYFP lines were performed using YFP/GFP antibodies in liverworts 24 hours after E (50 µM estradiol) or D (0.25% DMSO) in 3-4-week-old thalli. Ponceau staining indicated protein loading. Performed twice with similar results.

(D) RT-qPCR expression analysis of liverwort immune-associated genes in XVE:MLA10^{CC}-eYFP lines relative to an XVE:mCitrine-HA control. Thallus treatment was performed as described above, with RNA extracted 1 day post treatment. Transcripts were quantified relative to internal Mp*ACT* and Mp*EF1a* controls. Different letters signify statistically significant differences in transcript abundance (ANOVA, Tukey's HSD, $p < 0.05$). Values represent the mean +/- standard deviation of three biological replicates (n = 6). This experiment was performed three times with similar results.

A.

Organism	Comparison	Total DEGs	Orthologous DEGs	Remaining DEGs
Marchantia polymorpha	MpCNL1^{CC}-eYFP vs mCit-HA	1572	362	1210
Nicotiana benthamiana	MpCNL1^{CC}-eYFP vs GUS-YFP	5298	550	4748

B.

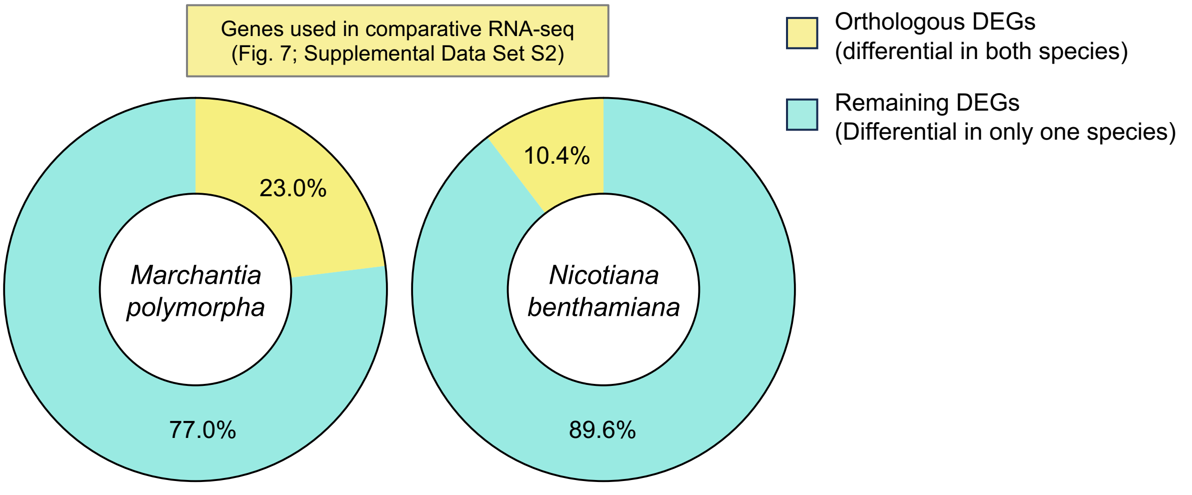

Supplemental Figure S11. Orthologous and differentially expressed genes used for comparative transcriptomics. Supports Figure 7

(A) Table outlining the organisms, treatments, and differentially expressed genes (DEGs) used for inter-lineage comparisons of CC-induced changes in host transcriptional profiles. Total DEGs per species, orthologous DEGs (differential in both species), and remaining DEGs that do not have differentially expressed counterparts between species are shown.

(B) Visual representation of the proportion of each transcriptome that is analysed in comparative analysis of orthologous DEGs. Values represent the percentage of each category relative to the total set of DEGs within the indicated species and are rounded to the nearest tenth.
