# Supplementary material for: The N-terminal domains of NLR immune receptors exhibit structural and functional similarities across divergent plant lineages
Source: Plant Cell. 2024 Apr 10;36(7):2491–511. doi: 10.1093/plcell/koae113 (PMC11218826; doi:10.1093/plcell/koae113)
Supplement: koae113_Supplementary_Data [file koae113_supplementary_data.zip › Supplemental File 3.pdf]

A.

Motif	LOGO	E-value	Sites	Width
1	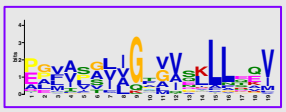	3.0e-352	80	19
2	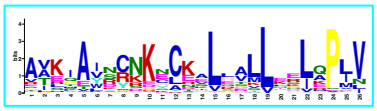	6.2e-749	84	26
3	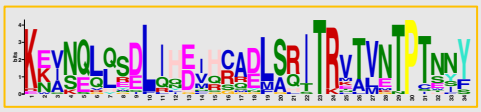	1.3e-346	22	34
4	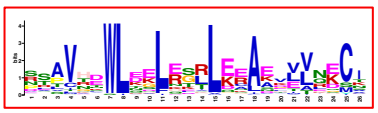	7.4e-741	83	26
5	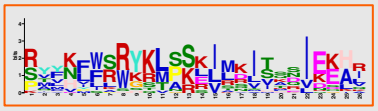	5.1e-236	33	26
6	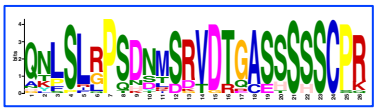	6.3e-284	20	26
7	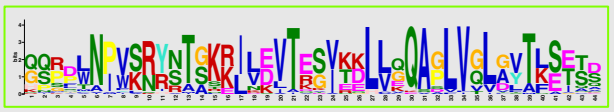	1.1e-837	39	44
8	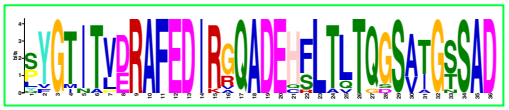	1.5e-331	14	36
9	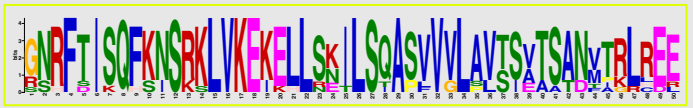	1.8e-223	10	50
10	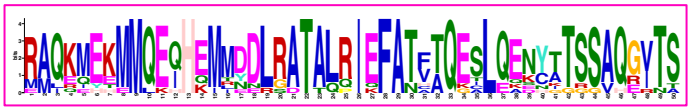	6.2e-314	12	50

Supplemental File 3. MAEPL motif discovery
(A) MEME analysis (MEMEsuite) using 105 OG6-type CC domains encoded within non-flowering land plants. The top ten amino acid motifs with “zero or one occurrence per sequence” are depicted. Width denotes size in amino acid residues.

B.

Motif 1

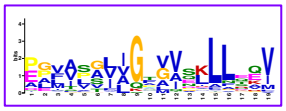

Motif 2

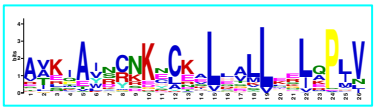

Motif 3

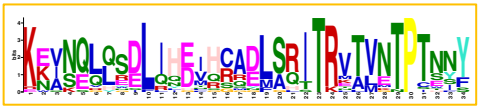

Motif 4

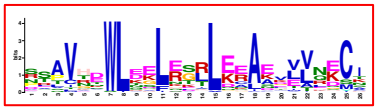

Motif 5

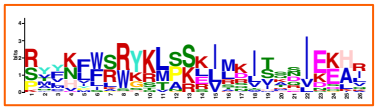

Motif 6

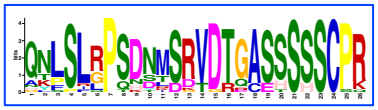

Motif 7

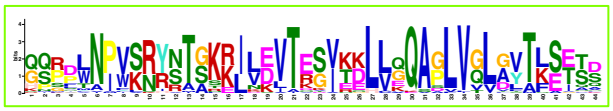

Motif 8

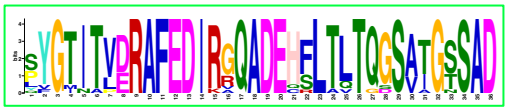

Motif 9

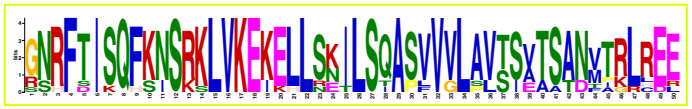

Motif 10

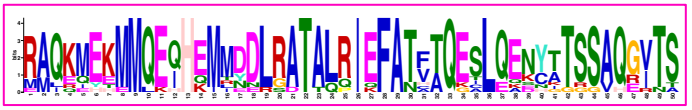

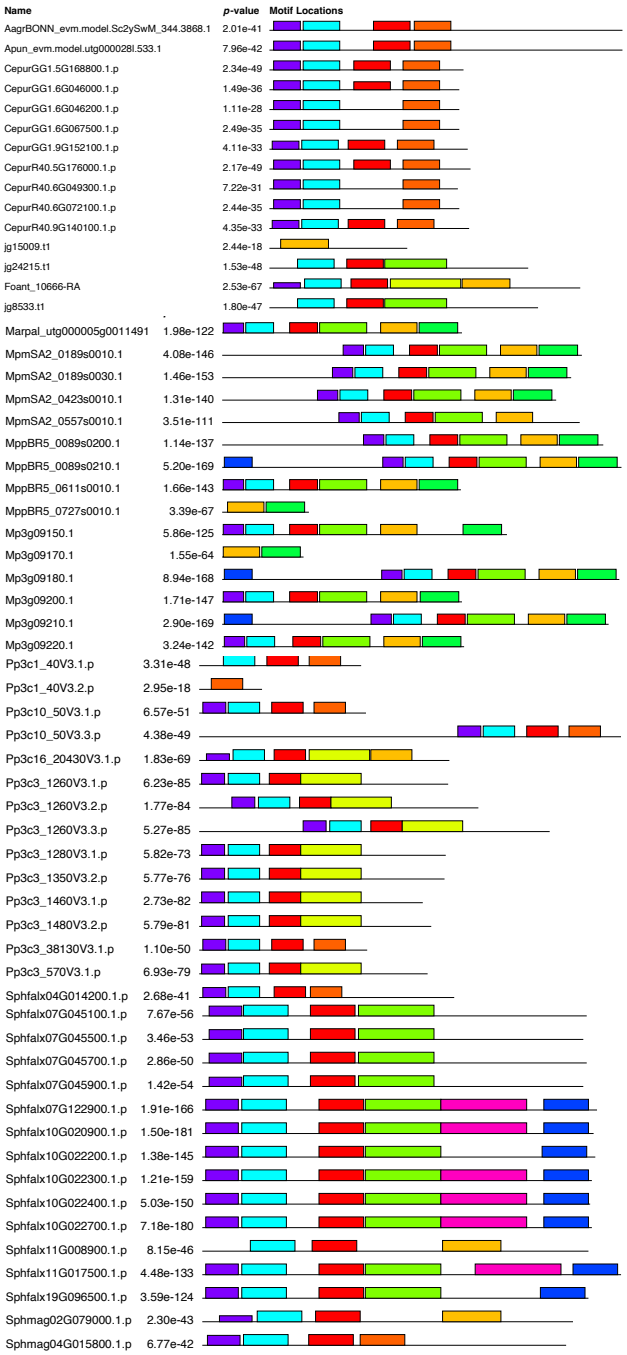

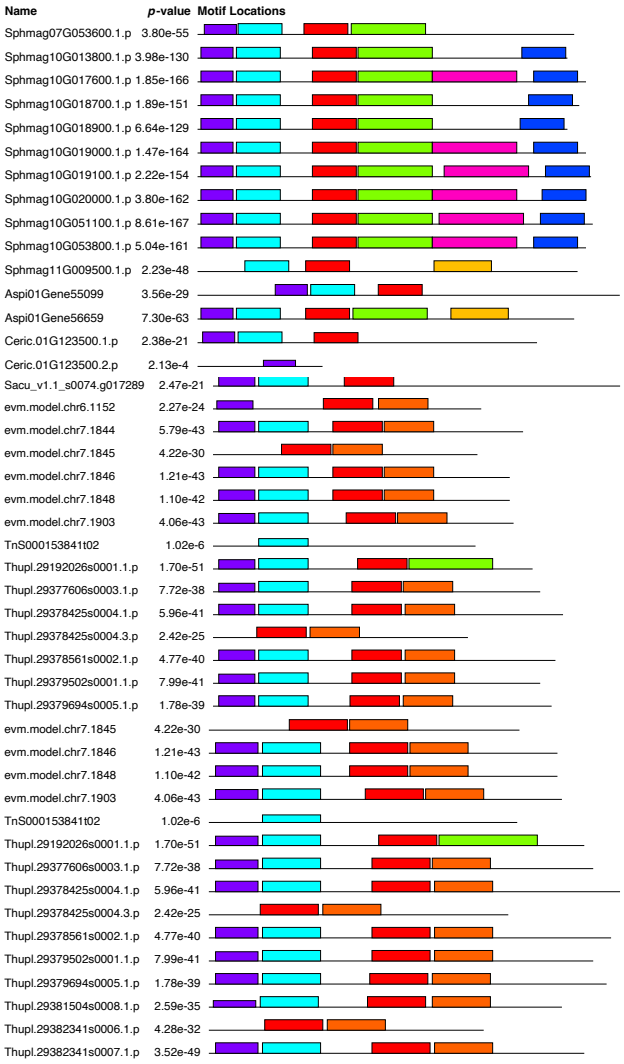

Supplemental File 3. MAEPL motif discovery
(B) Visual representations of motif location across 105 OG6-type CC domains.

C.

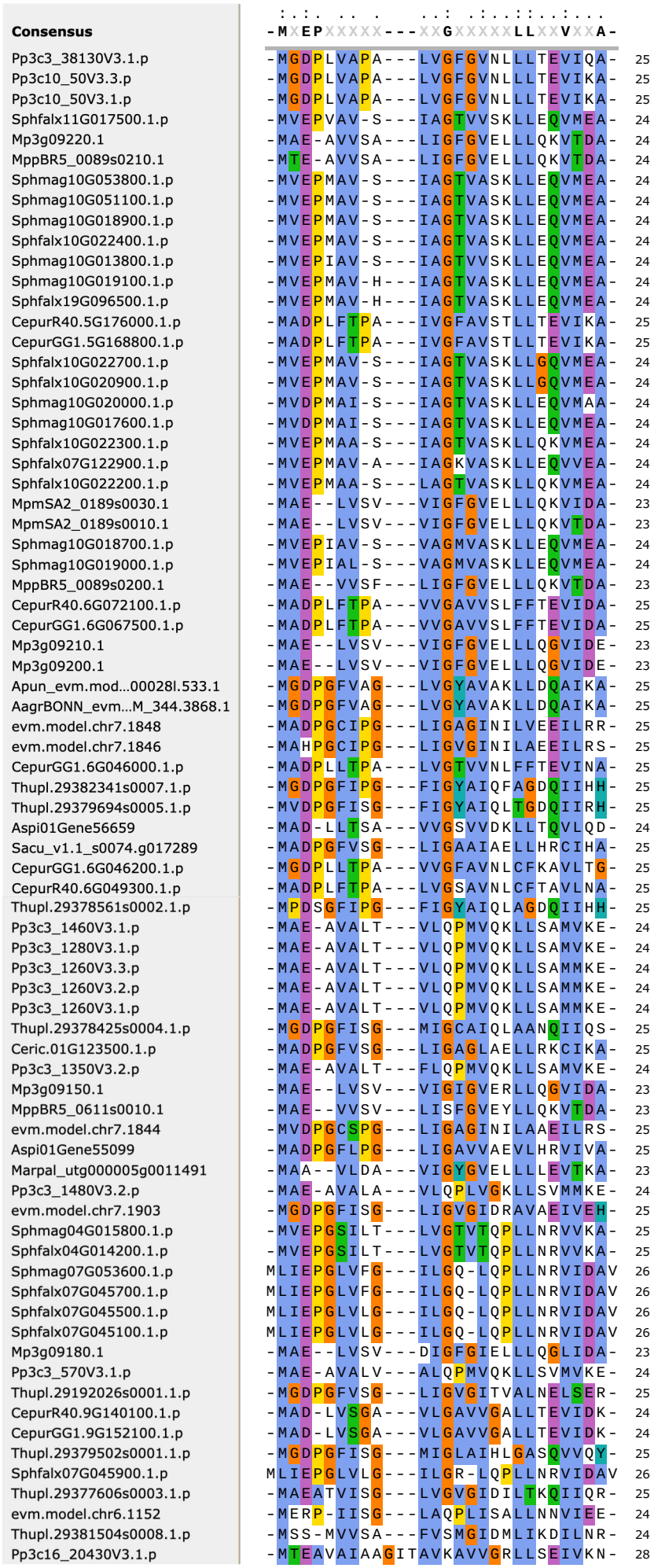

Supplemental File 3. MAEPL motif discovery

(C) Amino acid sequence alignment of the N-terminal MAEPL motif present in a subset of OG6-type CC domains of non-flowering plants. Alignments were performed using MAFFT in the SnapGene tool (v6.0.2). The consensus sequence shown represents residues present in >55% of all sequences. Coloring is based on amino acid residue properties + conservation (ClustalX).
