# Supplementary figures and images for: The N-terminal domains of NLR immune receptors exhibit structural and functional similarities across divergent plant lineages
Source: Plant Cell. 2024 Apr 10;36(7):2491–511. doi: 10.1093/plcell/koae113 (PMC11218826; doi:10.1093/plcell/koae113)

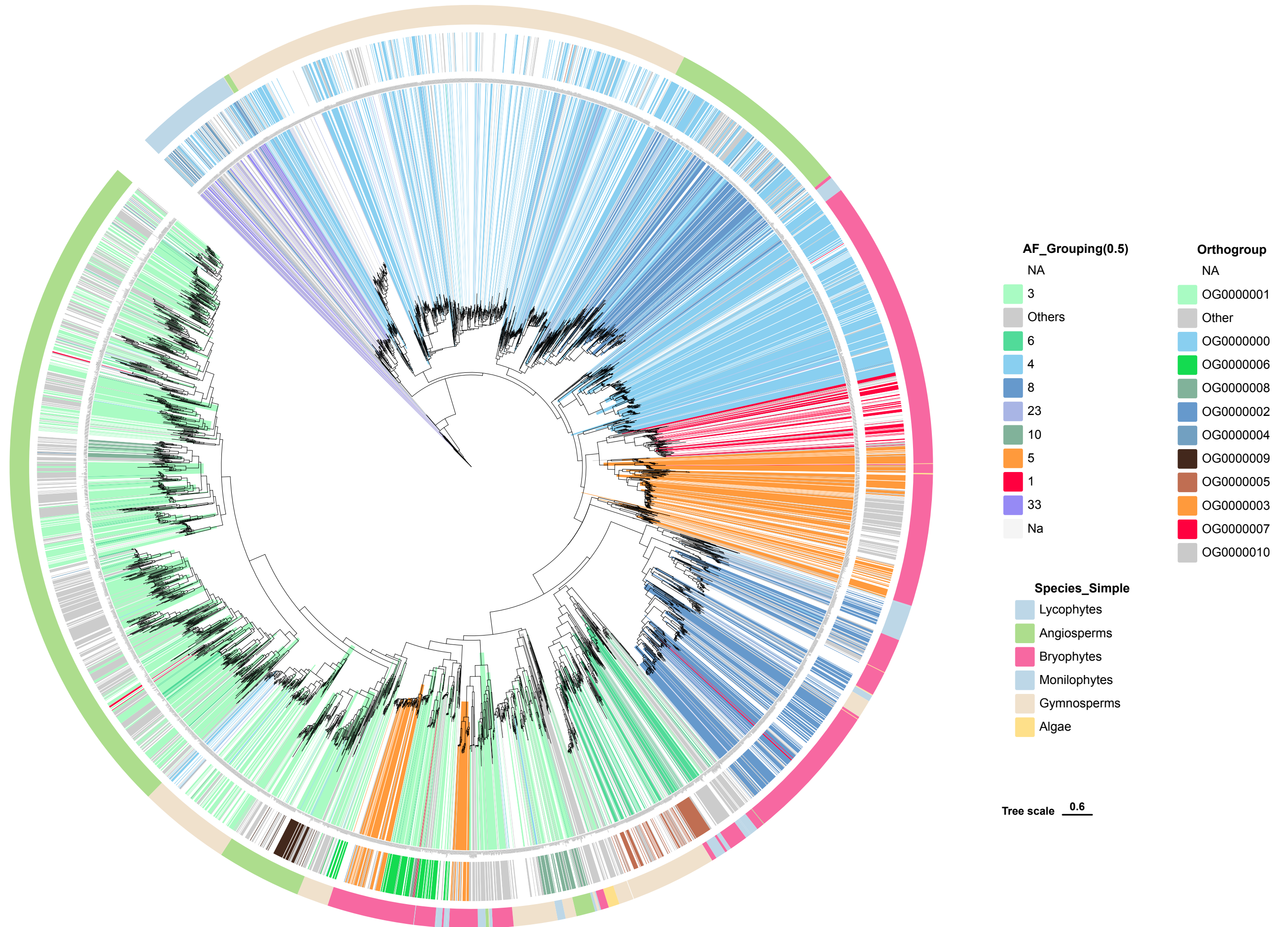

Supplement: koae113_Supplementary_Data [file koae113_supplementary_data.zip › Supplemental File 4.pdf]
