# Supplementary material for: The N-terminal domains of NLR immune receptors exhibit structural and functional similarities across divergent plant lineages
Source: Plant Cell. 2024 Apr 10;36(7):2491–511. doi: 10.1093/plcell/koae113 (PMC11218826; doi:10.1093/plcell/koae113)
Supplement: koae113_Supplementary_Data [file tpc.01017.2023-s12.pdf › tpc.01017.2023-s12.pdf]

THE PLANT CELL

¬The N-terminal domains of NLR immune receptors exhibit structural and functional similarities across divergent plant lineages

Khong-Sam Chia, Jiorgos Kourelis, Albin Teulet, Martin Vickers, Toshiyuki Sakai, Joseph Walker, Sebastian Schornack, Sophien Kamoun, and Philip Carella

Review Timeline:

Submission Date:	27-Oct-2023
Editorial Decision:	04-Dec-2023
Revision Received:	11-Mar-2024
Accepted:	18-Mar-2024

Dear Dr. Carella/Dear Phil:

We have received reviews of your manuscript entitled "The N-terminal executioner domains of NLR immune receptors are structurally and functionally conserved across divergent plant lineages." On the basis of the advice received, the board of reviewing editors would like to accept your manuscript for publication in The Plant Cell. This acceptance is contingent on revision based on the comments of our reviewers. In particular, please consider the following:

While all three reviewers concurred that this work provides significant new insight into the evolution of plant NLRs, reviewers 2 and 3 raised a number of questions that should be addressed in the revision, most of which can be addressed by clarifications in the text. The title should also be modified as suggested by Reviewer 1. In addition, we concur with reviewers 2 and 3 that the most novel aspect of this work is the identification of kinase and hydrolase N-terminal domains in the non-angiosperm NLRs and that this finding should be followed up by functional testing in N. benthamiana to assess whether dimerization/oligomerization of these domains can also elicit cell death.

Please highlight all changes and include a detailed annotation of changes of the text, with line numbers, and noting your responses to the comments.

To submit your revised manuscript, click:

Link Not Available

If you have any questions about the revision submission procedures, please contact the Editorial Office Staff (tpc-submissions@aspb.org). If you cannot return the revised manuscript within 30 days, please let us know. Otherwise, we will assume that you have elected not to revise the manuscript and withdraw it.

Thank you very much for the privilege of reviewing this work. I look forward to receiving the next version.

Sincerely,

The Plant Cell Editorial Board

(Please note that due to a potential conflict of interest, EIC Blake Meyers did not participate in evaluating this manuscript).

Reviewer #1 (Comments for the Author):

It was a pleasure to review the Chia et al. manuscript. It covers a long-awaited functional study of NLRs and N-terminal signaling domains outside Angiosperms. The entire manuscript was easy to read and had quality figures with individual data points displayed. I do not have major reservations about this manuscript. The data support all claims in the main text.

Still, I suggest making the title more specific, for instance, "N-terminal domain of intracellular immune receptor-like proteins from divergent plant lineages trigger cell death and transcriptional reprogramming". Why? First, the term "executioner domain" implies that it executes an immune response after the immune receptor activation, for which there is no published evidence that non-seed NLRs act as such. Second, "functionally conserved" seems too broad because the activation of Ca^{2+} influx or dependency of the TIR cell death on the EDS1/helper NLR was not assessed (which is not needed for the manuscript).

Other minor suggestions for improving the clarity:
L190-192: To which OG and AF groups do TNPs belong? You talk about this protein group in several locations of the manuscript, and I think it would help to show it in Fig 2D
L207-214: please specify in the Materials and Methods, Main text or Supplementary Notes how you denoted domain borders for the functional analysis. This information might help to understand why specific fragments did or did not cause cell death.
L332-392: I missed numbers of DEGs and tests for overlap between gene sets when the authors discuss (dis)similarities between MpC1 and other samples. Examples: "drastic change" (L338), "large set" (L340), "several" (L349), "strong" (L351, L369), missing ratio of up vs all genes (L371-372, not only rough %), "significant" (L374). How statistically significant is the overlap between DEGs in L371 and L381?
L477-480: the statements are too strong; please consider changing them. Based on the ontology enrichment analysis, authors proposed that cell death is caused by perturbed DNA replication and damage, and they later suggested that this is evolutionarily conserved. Authors say the latter is to be clarified in further research, while it seems necessary to establish the DNA damage - cell death link first.

Reviewer #2 (Comments for the Author):

All plant lineages are known to possess NLRs from algae to seed plants, but our understanding of functions of different classes of NLRs were mostly derived from those in higher plants. In this study, the authors compared different NLRs from lower plants to higher plants, and addressed whether different N terminal domains of lower plant NLRs function similarly as their higher plant counterparts. The authors queried 33 species to cover the entire green lineage and confirmed the presence of different N terminal domains in lower plant lineages. They further applied orthofinder and alphafold to annotate the N terminal domains into different OGs and AF clusters. Selected TIR and CC domains were tested for HR-induction, and many induced HR, which is similar to TIR and CC from higher plants. Interestingly, one of the OGs (OG6) is enriched in a motif the authors called MAEPL motif. MAEPL is similar to the previously reported MADA motif in amino acid sequence and functionality (functionally interchangeable). The authors further showed that MpCNL1 is functionally similar to MAD-motif-containing CNLs in that its ability to induce cell death and plasma membrane-localization in M patens depends on the N-terminal CC. Finally, the authors provide transcriptome data to support that MpCNL1 activate similar defenses as higher plant counterparts. Overall the manuscript shows that TIR and CC of plant NLRs are conserved in sequence, structure and function throughout the evolutionary history, the observations are expected but nonetheless useful. However, several questions need to be addressed prior to publication.

1. Only some TIRs and CCs trigger HR when overexpressed as YFP-fusion protein. We know not all NLRs execute signals. For instance, many sensor NLRs do not trigger HR by themselves. The manuscript does not provide information as to which clusters of OGs and AFs are enriched for HR-inducing activity. Is the selection of CCs and TIRs in Fig 4 random? Why not systematically test CCs and TIRs from different clusters of OGs and AFs?
2. OG6 is enriched for MAEPL-containing NLRs. What about OGs and AFs that are enriched for MADA motif? How robust are the OG and AF-based categorization in terms of prediction of functional groups. It is rather surprising (and interesting) that both ADR1 and ZAR1 belong to AF3. What about OG6? Does it also fall into AF3? OGs and AFs should be carefully compared and discussed in the context of their functions to be meaningful.
3. Hydrolase and kinase domains are unique to lower plant NLRs. It would be much more novel to address functionality of these NLRs than showing the expected function of TIR and CC domains.

Reviewer #3 (Comments for the Author):

The manuscript by Chia et al surveys N-terminal domains associated with NLR immune receptors in a selection of plant species. The authors test a selection of ~20 TIR domains and ~40 CC domains, but none of the identified hydrolase or kinase N-terminal domains, for expression and ability to induce HR in N. benthamiana as eYFP fusions (Figure 4). The paper then focuses on defining and characterizing a subset of CC domains present in non-flowering lineages that the authors name MAEPL after a 25-residue conserved sequence found in the very N-terminus of the CC domain, and conclude it is likely related by descent to previously described MADA-motif CCs.

Overall, the results are not entirely surprising and agree with the accepted view that N-terminal domains of NLRs are used to initiate downstream immune responses. This work is important because it describes NLR architectures found in non-flowering plants and has the potential to shed light on the early events in the evolution of the plant intracellular immune receptors. It is also timely, because of the recent genomics data that it draws upon.

Major concerns:
1. I would strongly advise using "signaling" as opposed to "executioner" in the title and elsewhere. Calcium fluxes by themselves do not execute immune functions, neither do diffusible signals downstream of TIR activity. EDS-1 dependence of many TNLs is a case in point.

2. The minimal representation of green algae is unfortunate: the methods developed to characterize N-termini (especially AF structure-based approach) could be very interesting when applied to a wider selection of early-branching species.

3. A question that is not directly addressed but would advance the field is which architectures were present in the common ancestor of green plants and which were derived later and when; including whether and when multiple TIR-, hydrolase-, kinase- , CC- containing NLR clades were independently derived. With that in mind, AF clusters seem somewhat arbitrary. If two clusters represent the same ancestral TNL architecture why not merge them? Otherwise, strange situations arise in Figure 3 where two different TIR clusters interdigitate in the RPS4 clade. I am also puzzled by the lack of links between AF-33 and the rest of TIR clades in F2D.

4. The model for AF-23 appears to not contain the central beta-sheet of TIR domain, are all AF-23 sequences similarly degenerate? Would it be possible based on what we know about TIR domain structure and function to sort AF prediction into likely functional/non-functonal?

5. I am surprised by OG4 and OG5 overlapping multiple AF clusters in F2E. What is the reason for this? Are these hitting the same underlying sequence or different subsequences?

6. I disagree with authors' interpretation that kinase and hydrolase architectures were "within a larger clade" of TNLs (line 187).

One would need to know where the root is in the tree to make this conclusion, and also that TNLs are monophyletic. For example, one of the TIR clades groups with AF6 Coiled-coil clade in the Figure 3 tree as best as I can tell.

7. Figure 3 colors are inconsistent making the figure hard to read. For example, orange shade is used for both CC and hydrolase domains. OG and AF colors should be fewer and matched based on 2E.

8. The iToL link listed in the manuscript does not contain the current version of Figure 3. The tree that is there has two sets of bootstrap values, which are not explained in text, and both appear high for the internal nodes on NB-ARC trees. The methods do not mention how many sequences were removed from consideration during the "cleaning" procedure on line 848? Were only terminal branches subject to removal? What would the authors loose by keeping these in? Were unique domain architectures eliminated as a result?

9. Arguably, for Figure 3, the most interesting regions encompass parts of the outer circle where the lineages are mixed downstream of a well-supported clade. This is evidence that supports these architectures being inherited from the common ancestor of the present species. I would appreciate a supplemental figure or a main subfigure zooming in on these ~6 areas that would include names of known genes and domain architectures in addition to the other annotation layers already present.

10. I believe that several hydrolase and kinase domains should be tested for HR using transient expression assay as these represent major new NLR clades.

11. Because eYFP is only weakly dimeric (Kofoed 2008), what is the authors' interpretation for why the HR assay works? (I think this should be discussed in the article main text.) Would HR phenotypes be observed upon inclusion of a strongly dimerizing coiled-coil C-terminal to the eYFP, such as the GCN4 leucine zipper, for inactive TIRs?

12. Both HR and WB assays lack a negative control such as eYFP alone. I would also appreciate an active site point mutant in one of the active TIRs to minimally test the conservation of the TIR mechanism.

13. Differential gene expression lacks statistical rigor and a clear null. Would we expect 50% concordant DEGs by random chance, are we surprised by 62%? Similarly for enrichment of specified groups of proteins in F7E (line 384), are the authors comparing presence in concordant vs discordant quadrants? Are they correcting for multiple testing given the large number of categories? How significant are the enrichment results?

Minor points:

1. Where are MADA and MAEPL CNLs found on the tree in Figure 3? It would make sense to highlight them to tie the two parts of the paper together better. It would also help a reader judge how likely these are to be related by descent.

2. Where are the TIR-NBARC-TPR NLR's that are mentioned in line 191 in Figure 3? Are they monophyletic with other TNLs?

3. There is an interspersed repeat of "to date" in line 47.

4. Both NBARC and NB-ARC appear in the main text.

Please also note the following:

-The Plant Cell now requires authors to complete and submit an author revisions checklist upon submission of a revised manuscript. The aim of the checklist is to aid authors in preparing a high-quality manuscript, facilitate the review and assessment of revised manuscripts, and help to ensure that journal standards are maintained across the board. If your manuscript is accepted, the completed checklist will be published as supplemental material attached to the article online. Please download a copy of the checklist (pdf fillable form) at this link, for submission with your revised manuscript: https://tpc.msubmit.net/html/Author_Revisions_Checklist.pdf.

-Supplemental materials should be restricted to large datasets and tables, presentation of replicates, and validation of reagents, methods, or genotypes. Any data that are used to support the major claims must be in the main manuscript. Supplemental figure legends must indicate what figure in the main manuscript is supported by the supplemental data presented. Please justify how each of the supplemental figures meet the criteria.

-Sampling methods and the nature of "biological replicates" should be described precisely (i.e. different plants, parts of plants, pooled tissue, independent pools of tissue, sampled at different times, etc.), along with a clear description of and rationale for any statistical analyses conducted. The reader should know exactly what was sampled; what forms the basis of the calculation of any means and statistical parameters reported. This is also necessary to ensure that proper statistical analysis was conducted.

Dear Dr. Carella/Dear Phil:

We have received reviews of your manuscript entitled "The N-terminal executioner domains of NLR immune receptors are structurally and functionally conserved across divergent plant lineages." On the basis of the advice received, the board of reviewing editors would like to accept your manuscript for publication in The Plant Cell. This acceptance is contingent on revision based on the comments of our reviewers. In particular, please consider the following:

While all three reviewers concurred that this work provides significant new insight into the evolution of plant NLRs, reviewers 2 and 3 raised a number of questions that should be addressed in the revision, most of which can be addressed by clarifications in the text. The title should also be modified as suggested by Reviewer 1. In addition, we concur with reviewers 2 and 3 that the most novel aspect of this work is the identification of kinase and hydrolase N-terminal domains in the non-angiosperm NLRs and that this finding should be followed up by functional testing in N. benthamiana to assess whether dimerization/oligomerization of these domains can also elicit cell death.

Please highlight all changes and include a detailed annotation of changes of the text, with line numbers, and noting your responses to the comments.

We thank the editors and reviewers for their encouragement and support. Please find our responses to reviewer comments below in ***blue*** text.

To submit your revised manuscript, click:

https://tpc.msubmit.net/cgi-bin/main.plex?el=A7Is2NfH6A6iQf6I6A9ftdSv2o9GT5y11TJuQ3e4sULgZ

If you have any questions about the revision submission procedures, please contact the Editorial Office Staff (tpc-submissions@aspb.org). If you cannot return the revised manuscript within 30 days, please let us know. Otherwise, we will assume that you have elected not to revise the manuscript and withdraw it.

Thank you very much for the privilege of reviewing this work. I look forward to receiving the next version.

Sincerely,

Thee Plant Cell Editorial Board

(Please note that due to a potential conflict of interest, EIC Blake Meyers did not participate in evaluating this manuscript).

Reviewer #1 (Comments for the Author):

It was a pleasure to review the Chia et al. manuscript. It covers a long-awaited functional study of NLRs and N-terminal signaling domains outside Angiosperms. The entire manuscript was easy to read and had quality figures with individual data points displayed. I do not have major reservations about this manuscript. The data support all claims in the main text.

Still, I suggest making the title more specific, for instance, "N-terminal domain of intracellular immune receptor-like proteins from divergent plant lineages trigger cell death and transcriptional reprogramming". Why? First, the term "executioner domain" implies that it executes an immune response after the immune receptor activation, for which there is no published evidence that non-seed NLRs act as such. Second, "functionally conserved" seems too broad because the activation of Ca2+ influx or dependency of the TIR cell death on the EDS1/helper NLR was not assessed (which is not needed for the manuscript).

We thank the reviewer for their encouraging assessment and support. We agree with this reviewer and others about altering the title. Since we would like to keep the structure/function aspect of the previous title we propose the following:

The N-terminal domains of NLR immune receptors exhibit structural and functional similarities across divergent plant lineages

Other minor suggestions for improving the clarity:
L190-192: To which OG and AF groups do TNPs belong? You talk about this protein group in several locations of the manuscript, and I think it would help to show it in Fig 2D

TNPs were included in a previous version of this manuscript (bioRxiv version) but were removed from the phylogeny during our unbiased cleaning processes (please see response to Reviewer 3 point 8). We apologize for the oversight and now remove references to TNPs in the results since we could not explore these further.

L207-214: please specify in the Materials and Methods, Main text or Supplementary Notes how you denoted domain borders for the functional analysis. This information might help to understand why specific fragments did or did not cause cell death.

CC, TIR, and Pkn domains were synthesized up to (but not including) NB-ARC start sites, whereas *Marchantia* Hyd domains were synthesized up to the middle of an alpha helix that links hydrolases to the NB-ARC. We have clarified this in the materials method (lines 796-800) and provide full sequences of all domains tested (Data S6, sheet 2).

L332-392: I missed numbers of DEGs and tests for overlap between gene sets when the authors discuss (dis)similarities between MpC1 and other samples. Examples: "drastic change" (L338), "large set" (L340), "several" (L349), "strong" (L351, L369), missing ratio of up vs all genes (L371-372, not only rough %), "significant" (L374). How statistically significant is the overlap between DEGs in L371 and L381?

We have adjusted the text throughout this section to provide further clarity on numbers of DEGs/ratios, and to tone down statements referring to ‘strength’ or ‘significance’. The first sentences in this section refer to the heatmaps depicted in Figure 7A, which visually demonstrate overlapping expression profiles. Next, we described sets of differentially expressed genes (DEGs) that are represented by Venn diagrams in Fig 7B (and in Fig S8 - previous Fig S5) and as requested we now include total numbers of DEGs for each condition directly in the text (line 369-70). We also indicate the precise number of upregulated CC-NLRs (6) on line 377. In addition, we have rephrased the sentence describing the overlap

observed between *N. benthamiana* genes activated by MLA10 and MpC1 to be more precise (line 399).

In this study, our focus was to identify and characterize orthologous DEGs shared between Marchantia and Nicotiana to begin to unravel common molecular responses to CC domain activity. We now clarify this in the text (line 404) and provide additional information on the proportion of the Marchantia and Nicotiana transcriptomes being analysed when focusing only on shared DEGs (i.e the numbers of shared orthologous DEGs -vs- total DEGS) (Figure S10; lines 408-11).

L477-480: the statements are too strong; please consider changing them. Based on the ontology enrichment analysis, authors proposed that cell death is caused by perturbed DNA replication and damage, and they later suggested that this is evolutionarily conserved. Authors say the latter is to be clarified in further research, while it seems necessary to establish the DNA damage – cell death link first.

We have toned down statements on the speculated function of DEGs in the results and discussion sections as suggested by the reviewer. We have also rephrased this speculation to include the possibility that DNA damage may be general a consequence of cell death (lines 505-7).

Reviewer #2 (Comments for the Author):

All plant lineages are known to possess NLRs from algae to seed plants, but our understanding of functions of different classes of NLRs were mostly derived from those in higher plants. In this study, the authors compared different NLRs from lower plants to higher plants, and addressed whether different N terminal domains of lower plant NLRs function similarly as their higher plantcounterparts. The authors queried 33 species to cover the entire green lineage and confirmed the presence of different N terminal domains in lower plant lineages. They further applied orthofinder and alphafold to annotate the N terminal domains into different OGs and AF clusters. Selected TIR and CC domains were tested for HR-induction, and many induced HR, which is similar to TIR and CC from higher plants. Interestingly, one of the OGs (OG6) is enriched in a motif the authors called MAEPL motif. MAEPL is similar to the previously reported MADA motif in amino acid sequence and functionality (functionally interchangeable). The authors further showed that MpCNL1 is functionally similar to MAD-motif-containing CNLs in that its ability to induce cell death and plasma membrane-localization in M patens depends on the N-terminal CC. Finally, the authors provide transcriptome data to support that MpCNL1 activate similar defenses as higher plant counterparts. Overall the manuscript shows that TIR and CC of plant NLRs are conserved in sequence, structure and function throughout the evolutionary history, the observations are expected but nonetheless useful. However, several questions need to be addressed prior to publication.

1. Only some TIRs and CCs trigger HR when overexpressed as YFP-fusion protein. We know not all NLRs execute signals. For instance, many sensor NLRs do not trigger HR by themselves. The manuscript does not provide information as to which clusters of OGs and AFs are enriched for HR-inducing activity. Is the selection of CCs and TIRs in Fig 4 random? Why not systematically test CCs and TIRs from different clusters of OGs and AFs?

The selection of domains in Figure 4 stemmed from efforts to characterize a representative set of domains for each plant lineage (especially those from model bryophytes like Marchantia and Physcomitrium). We provide summary information on the identity (OG/AF) and function of all tested domains in Data S1 (sheet 6), which now includes additional atypical hydrolase (OG3/AF5) and kinase (OG7/AF1) domains (Figure S4). We then focus our attention to a prominent group of non-flowering plant CC domains (OG6/AF3) and dissect their function for the first time in liverworts and in the angiosperm Nicotiana.

2. OG6 is enriched for MAEPL-containing NLRs. What about OGs and AFs that are enriched for MADA motif? How robust are the OG and AF-based categorization in terms of prediction of functional groups. It is rather surprising (and interesting) that both ADR1 and ZAR1 belong to AF3. What about OG6? Does it also fall into AF3? OGs and AFs should be carefully compared and discussed in the context of their functions to be meaningful.

A comparison between sequence groups (OGs) and structure clusters (AFs) is presented in Figure 3E. This heatmap shows the overlap between the major sequence/structure groups and demonstrates that (a) some groups are tightly overlapping in sequence and predicted structures (e.g. kinases or hydrolases), or (b) that sequence diverse OGs can fall into a smaller number of AF structure clusters. The latter is particularly evident for CC and RPW8 domains that are sorted into a large number of OG sequence groups but converge into a much fewer AF clusters. This makes sense since angiosperm CCs and RPW8s share a common 4 helix bundle, which is similar to that of the OG6 CC domain and occurs across several groups irrespective of their functional capacities. While a systematic analysis of the thousands of N-terminal domain groups is beyond the scope of our initial study, we do provide evidence that the CC-OG6 domains of non-flowering plants shares structural similarity to functional CC/RPW8 domains of angiosperms and that it encodes an N-terminal motif (MAEPL) that is functionally equivalent to the angiosperm MADA motif. An improved CC/RPW8 phylogeny showing the location of MADA-vs-MAEPL motifs is shown in Data S4E.

3. Hydrolase and kinase domains are unique to lower plant NLRs. It would be much more novel to address functionality of these NLRs than showing the expected function of TIR and CC domains.

We now include functional data of hydrolase and kinase domains transiently expressed in *N. benthamiana* (Figure S4).

Reviewer #3 (Comments for the Author):

The manuscript by Chia et al surveys N-terminal domains associated with NLR immune receptors in a selection of plant species. The authors test a selection of ~20 TIR domains and ~40 CC domains, but none of the identified hydrolase or kinase N-terminal domains, for expression and ability to induce HR in N. benthamiana as eYFP fusions (Figure 4). The paper then focuses on defining and characterizing a subset of CC domains present in non-flowering lineages that the authors name MAEPL after a 25-residue conserved sequence found in the very N-terminus of the CC domain, and conclude it is likely related by descent to previously described MADA-motif CCs.

Overall, the results are not entirely surprising and agree with the accepted view that N-terminal domains of NLRs are used to initiate downstream immune responses. This work is important because it describes NLR architectures found in non-flowering plants and has the

potential to shed light on the early events in the evolution of the plant intracellular immune receptors. It is also timely, because of the recent genomics data that it draws upon.

Major concerns:

1. I would strongly advise using "signaling" as opposed to "executioner" in the title and elsewhere. Calcium fluxes by themselves do not execute immune functions, neither do diffusible signals downstream of TIR activity. EDS-1 dependence of many TNLs is a case in point.

We appreciate the reviewer's position and we have removed 'executioner' from much of the manuscript (including the title and figure legends). However, we do not feel comfortable labelling CC/RPW8 domains as 'signaling' as they directly participate in membrane pore formation that is associated with confluent cell death across many systems. On line 61 we now describe N-terminal domains as *'executioner' or 'signaling'* domains and expose the reader to both options.

2. The minimal representation of green algae is unfortunate: the methods developed to characterize N-termini (especially AF structure-based approach) could be very interesting when applied to a wider selection of early-branching species.

We share the author's view that the NLRs of green algae are an interesting avenue of investigation and we made sure to include TIRs and CCs derived from these organisms in our functional assessment of N-terminal domains (Figure 4). We demonstrate that their N-terminal domains (only CC and TIRs were detected) are functionally transferable to an angiosperm system, which addresses a key question on NLR functional evolution. We hope to leverage emerging algal genomes and model systems in our future work.

3. A question that is not directly addressed but would advance the field is which architectures were present in the common ancestor of green plants and which were derived later and when; including whether and when multiple TIR-, hydrolase-, kinase- , CC-containing NLR clades were independently derived. With that in mind, AF clusters seem somewhat arbitrary. If two clusters represent the same ancestral TNL architecture why not merge them? Otherwise, strange situations arise in Figure 3 where two different TIR clusters interdigitate in the RPS4 clade. I am also puzzled by the lack of links between AF-33 and the rest of TIR clades in F2D.

We agree that this is an interesting idea, however we believe it is outside of the scope of our study on the structural and functional similarities of plant NLR N-terminal domains. Future studies incorporating additional algae (streptophytes and chlorophytes) will be of particular interest but require considerable work to address properly. AF clustering was performed based strictly on structural similarity, therefore we refrained from merging groups. The interdigitation of certain clades indicates structural differences that were sensitive enough to be discovered by clustering analyses, which could be explained by domain truncations or additions (additional integrated domains, or other insertions). We focused our attention on broadly distributed N-terminal domains and therefore did not investigate the AF33 clade further, though it is an interesting route of future investigation.

4. The model for AF-23 appears to not contain the central beta-sheet of TIR domain, are all AF-23 sequences similarly degenerate? Would it be possible based on what we know about TIR domain structure and function to sort AF prediction into likely functional/non-functonal?

Since we did not AF23 directly in our study we refrained from speculating about it's function. However, we now mention in the text that AF23 is scattered amongst larger clades of TIRs, suggesting that this may not be a distinct subclass of TIR-NLRs (line 201-3).

5. I am surprised by OG4 and OG5 overlapping multiple AF clusters in F2E. What is the reason for this? Are these hitting the same underlying sequence or different subsequences?

OG5 overlaps with the AF3 (CC) and AF6 (CC-like) structure clusters that each contain 4-helix bundles (AF6 can have additional helices) which explains the partial overlap between structure group assignments. For OG4, this is less clear, however the overlap implies that structural features common between TIRs and CCs (likely helices) are being detected. While we agree that this is interesting, we feel that further investigations of N-terminal domain structure are better suited for more detailed future studies.

6. I disagree with authors' interpretation that kinase and hydrolase architectures were "within a larger clade" of TNLs (line 187). One would need to know where the root is in the tree to make this conclusion, and also that TNLs are monophyletic. For example, one of the TIR clades groups with AF6 Coiled-coil clade in the Figure 3 tree as best as I can tell.

We have modified the text to better clarify the relationships between plant NLRs. This is clarified on lines 184-203. We also provide additional representations of the NB-ARC phylogeny to better highlight the main groups of NLRs (please see our response to point 9, below).

7. Figure 3 colors are inconsistent making the figure hard to read. For example, orange shade is used for both CC and hydrolase domains. OG and AF colors should be fewer and matched based on 2E.

We thank the reviewer for identifying this issue. The CC-OG6 group has been changed to a green color that contrasts against other CC groups (shades of green). The Hydrolase OG group is now the same orange color of the corresponding AF cluster. These modifications can be seen in Figure 3, Figure S2, and Dataset S4E.

8. The iToL link listed in the manuscript does not contain the current version of Figure 3. The tree that is there has two sets of bootstrap values, which are not explained in text, and both appear high for the internal nodes on NB-ARC trees. The methods do not mention how many sequences were removed from consideration during the "cleaning" procedure on line 848? Were only terminal branches subject to removal? What would the authors loose by keeping these in? Were unique domain architectures eliminated as a result?

We apologize for our oversight on the erroneous iTol link and we now provide the correct high resolution PDF of the tree displayed in Figure 3 as a supporting file. As requested, we also provide closer looks at the "CNL/RPW8" and "TIR/HYD/Kinase" areas of the phylogeny (Figure S2). The phylogeny in Figure 3 contains only a single bootstrap value (ultrafast bootstrap – UFboot). In addition, the methods section now includes information on the numbers of non-redundant NB-ARC sequences that were removed during the cleaning

procedure (252 cleaned, 4624 unique sequences remaining: lines 887-88) and we provide an additional table in Data S1 (sheet 10) that provides a thorough examination of the removed NB-ARCs and their corresponding architectures, AF clusters, and OGs. Overall, cleaned sequences belonged to diverse NLRs and NLR-like proteins that included TIR-Ploop, TIR-Ploop-TPR (TNPs), Hyd-NLRs, CC-NLRs, and various orphan NB-ARCs.

The reviewer is correct, only terminal branches were subject to removal. Maintaining those sequences in the tree can lead to false positives and having long branches can result in incorrect relationships away from those branches being inferred (https://doi.org/10.1093/sysbio/sys078). We now clarify this in the materials and methods (line 892).

9. Arguably, for Figure 3, the most interesting regions encompass parts of the outer circle where the lineages are mixed downstream of a well-supported clade. This is evidence that supports these architectures being inherited from the common ancestor of the present species. I would appreciate a supplemental figure or a main subfigure zooming in on these ~6 areas that would include names of known genes and domain architectures in addition to the other annotation layers already present.

We now highlight the major CNL/RNL clade by collapsing all other parts of the tree (Figure S2A), and we highlight the major 'TNL/HydNL/PknNL' regions of the phylogeny by collapsing the CNL/RNL clade (Figure S2B). We include names of known NLRs from angiosperms as well as key areas of interest that are outlined in the results (lines 184-203). We agree with the reviewer that this facilitates a better understanding of specific subclades and thank them for the suggestion.

10. I believe that several hydrolase and kinase domains should be tested for HR using transient expression assay as these represent major new NLR clades.

We now include this data in Figure S4 and the results are described in the text on lines 237-54.

11. Because eYFP is only weakly dimeric (Kofoed 2008), what is the authors' interpretation for why the HR assay works? (I think this should be discussed in the article main text.) Would HR phenotypes be observed upon inclusion of a strongly dimerizing coiled-coil C-terminal to the eYFP, such as the GCN4 leucine zipper, for inactive TIRs?

The use of YFP is common for NLR N-terminal domains and has been shown to enable cell death for CC domains, a 29 amino acid N-terminal MADA fragment, and for TIRs. Many N-terminal domains of angiosperms are also capable of self-association and don't necessarily require YFP for their function. We mention this point in the introduction on line 63. Since the exploration of CC-mediated responses in non-flowering plants (liverworts) is a key aspect of our work, we proceed with eYFP fusions that work well with angiosperm CCs. Re-testing each non-functional domain with a YFP-zipper is beyond the scope of this article, though it is an intriguing idea and could be the basis for future tool development.

12. Both HR and WB assays lack a negative control such as eYFP alone. I would also appreciate an active site point mutant in one of the active TIRs to minimally test the conservation of the TIR mechanism.

As a negative control, a GUS-eYFP fusion construct is included in every agroinfiltration experiment that we perform in the lab. We have updated the methods section to clarify this point (line 756-57). Data for the GUS-eYFP control is shown in Figure 7B, and in Figure S7D (previously Figure S4D). We now also include GUS-eYFP in our phenotyping of the hydrolase and kinase N-terminal domains (Figure S4), where again it shows a null phenotype.

We thank the reviewer for their suggestion to test active site mutants of TIR domains. In Figure S3 we demonstrate that the cell death phenotypes of AtRPS4 (control) and two non-flowering TIR domains all require the catalytic glutamic acid residue in N. benthamiana (lines 220-23).

13. Differential gene expression lacks statistical rigor and a clear null. Would we expect 50% concordant DEGs by random chance, are we surprised by 62%? Similarly for enrichment of specified groups of proteins in F7E (line 384), are the authors comparing presence in concordant vs discordant quadrants? Are they correcting for multiple testing given the large number of categories? How significant are the enrichment results?

We have toned down the language in this section, clarified the comparisons being made, and outline the extent to which orthologous DEGs are shared between Marchantia and Nicotiana through changes in the text and an additional supplementary figure (Figure S10; lines 408-11). We specifically selected shared orthologous DEGs in our analysis as we sought to define the common molecular responses caused by CC activity in two divergent plants. Functional enrichment analysis was performed using the online MBEX platform (https://doi.org/10.1093/pcp/pcac129) as described in the methods. We now include ‘functional enrichment’ directly in the title of this section to better direct the reader to this methodology. Functional enrichment analysis performed on MBEX detects over- or under-represented terms within a list of gene identifiers using Fisher’s Exact Test with p-values corrected by FDR (false discovery rate). Enrichment was considered for terms having FDR corrected p-values < 0.05 (all results and corresponding p-values are in Data S5, sheet 8). The figure legend for Fig 7E indicates that enrichment analyses were performed individually for each sector.

Minor points:

1. Where are MADA and MAEPL CNLs found on the tree in Figure 3? It would make sense to highlight them to tie the two parts of the paper together better. It would also help a reader judge how likely these are to be related by descent.

This information is included in the supporting data (Data S4E), which we mention this in the text on line 276.

2. Where are the TIR-NBARC-TPR NLR's that are mentioned in line 191 in Figure 3? Are they monophyletic with other TNLs?

We thank the reviewer for bringing this to our attention. The TNPs were present in our initial phylogeny (iTOL) but were mostly removed during the cleaning process. We have corrected the text to reflect this point, and we apologize for the confusion.

3. There is an interspersed repeat of "to date" in line 47.

Fixed, thank you for identifying this.

4. Both NBARC and NB-ARC appear in the main text.

We now use 'NB-ARC' throughout the manuscript, thank you for identifying this.

Please also note the following:

-The Plant Cell now requires authors to complete and submit an author revisions checklist upon submission of a revised manuscript. The aim of the checklist is to aid authors in preparing a high-quality manuscript, facilitate the review and assessment of revised manuscripts, and help to ensure that journal standards are maintained across the board. If your manuscript is accepted, the completed checklist will be published as supplemental material attached to the article online. Please download a copy of the checklist (pdf fillable form) at this link, for submission with your revised manuscript: https://tpc.msubmit.net/html/Author_Revisions_Checklist.pdf.

Complete and attached.

-Supplemental materials should be restricted to large datasets and tables, presentation of replicates, and validation of reagents, methods, or genotypes. Any data that are used to support the major claims must be in the main manuscript. Supplemental figure legends must indicate what figure in the main manuscript is supported by the supplemental data presented. Please justify how each of the supplemental figures meet the criteria.

Figure S1: Data justifying the use of techniques important for Figure 2
Figure S2: Additional phylogenies highlighting important aspects of Figure 3
Figure S3: Small follow up experiment related to Figure 4
Figure S4: Initial characterization of atypical domains, related to Figure 3/4
Figure S5: Computational analysis supporting main message of Figure 5
Figure S6: Small follow up experiment & replicate experiment related to Figure 5 and 6.
Figure S7: Extended phenotyping and troubleshooting of phenotypes describe in Figure 6
Figure S8: Comprehensive analysis and summary of RNA-seq datasets shown in Figure 7
Figure S9: Small supporting experiment justifying experimental comparison in Figure 7
Figure S10: Summary statistics and visual guide to experimental designs in Figure 7

Data S1: Computational dissection of NLR N-terminal diversity. Related to Figures 1-3.
Data S2: Cell death phenotyping of N-terminal domain in Nicotiana. Related to Figure 4.
Data S3: Protein immunoblots related to main and supporting figures.
Data S4: MEME analysis, alignments, supporting the MAEPL motif. Related to Figure 5.
Data S5: RNA-seq data related to Figure 7 and Figure S9
Data S6: Primers and synthesised gene constructs related to main and supporting figures
Data S7: Values and statistics of analyses presented in main and supporting figures

-Sampling methods and the nature of "biological replicates" should be described precisely (i.e. different plants, parts of plants, pooled tissue, independent pools of tissue, sampled at different times, etc.), along with a clear description of and rationale for any statistical analyses conducted. The reader should know exactly what was sampled; what forms the basis of the calculation of any means and statistical parameters reported. This is also necessary to ensure that proper statistical analysis was conducted.
Described in methods and figure legends where necessary.

Dear Dr. Philip Carella:

We are pleased to inform you that your paper entitled "The N-terminal domains of NLR immune receptors exhibit structural and functional similarities across divergent plant lineages" has been accepted for publication in The Plant Cell, pending a final editorial review by a science editor. At this stage, your manuscript will be evaluated by a science editor with respect to its presentation of scientific content, compliance with journal policies, and presentation for a broad readership. The Plant Cell has contracted with Plant Editors (planteditors.com) to provide this service to our authors, and you will soon receive additional information on this process.

Please note that each author needs to link their ORCID identifier to their account in the system before your manuscript can be published. If any authors do not have an ORCID linked to their account, they will receive a message with a link to complete this task. Please ensure that ALL of your coauthors have completed this task as soon as possible.

ASPB offers an OPEN option that allows authors to have their online articles available for free to all users immediately upon publication. For more information about the ASPB OPEN option, refer to the Final Submission Checklist Form.

The Plant Cell and The Arabidopsis Information Resource (TAIR) are collaborating to collect functional annotation data about Arabidopsis genes from authors. This includes information about the gene's molecular function (e.g., kinase activity, ATP synthetase activity), the biological process/es it is involved in (e.g., endosperm development, threonine biosynthesis), its subcellular location (e.g., nucleus, ER), anatomical or developmental expression pattern (e.g., leaf, ovule, flower stage 10, seedling stage), or its partner in a protein-protein interaction (e.g., AT1G01010 interacts with AT1G01020).

If your paper contains results falling into one or more of these categories for Arabidopsis genes, we request that you now submit these data for inclusion in TAIR by filling in the form provided at the following URL: https://www.arabidopsis.org/doc/submit/functional_annotation/123. If you need further clarification on what types of data can be submitted please contact curator@arabidopsis.org.

Finally, we encourage your submission of artwork for the journal cover. Monthly issues will have an online cover image and selected covers will be used for posters, other promotional items, and "wallpaper" for mobile devices. For more information, refer to Cover Submission in the Instructions for Authors https://tpc.msubmit.net/cgi-bin/main.plex?form_type=display_auth_instructions.

We look forward to seeing your paper published.

Sincerely,

The Plant Cell Editorial Board

(Please note that due to a potential conflict of interest, EIC Blake Meyers did not participate in evaluating this manuscript).

=====
IMPORTANT REMINDER: PEER REVIEW REPORTS
=====

If you opted to publish a peer review report along with your article during the original submission process, it will be prepared by the editorial staff and publicly posted with your manuscript, inside the zip file that contains any other supplemental material. As a reminder, the peer review report is a public record of all comments from editors and reviewers, as well as your prior responses, as you received them in the decision letters for each draft of your manuscript. If you agreed to publish this report and have changed your mind, or are not sure if you selected this option, please contact the editorial office as soon as possible before signing the license agreement from our publisher.

----FOR ASPB OFFICE USE ONLY (DO NOT EDIT)----
MSID: 36771
Corresponding Author: Dr. Carella
